# Factors associated with preoperative health-related quality of life in patients undergoing lumbar spine surgery: a multi-ethnic Asian cohort

**DOI:** 10.1007/s11136-026-04257-1

**Published:** 2026-05-03

**Authors:** Xun Li, Calvin Wei Jie Chern, Alex Quok An Teo, Jiong Hao Jonathan Tan, Annushiah Vasan Thakumar, Nan Luo, Hwee Weng Dennis Hey, Ling Jie Cheng

**Affiliations:** 1 School of Engineering, Computing, and Mathematics, Oxford Brookes University, Oxford, UK; 2 Alice Lee Centre for Nursing Studies, Yong Loo Lin School of Medicine, National University of Singapore, Singapore, Singapore; 3 Department of Orthopaedic Surgery, National University Hospital, Singapore, Singapore; 4 Department of Orthopaedic Surgery, Alexandra Hospital, Singapore, Singapore; 5 Yong Loo Lin School of Medicine, National University of Singapore, Singapore, Singapore; 6 School of Pharmacy, Faculty of Health & Medical Sciences, Taylor’s University, Subang Jaya, Selangor Malaysia; 7 Saw Swee Hock School of Public Health, National University of Singapore, Singapore, Singapore; 8 National Perinatal Epidemiology Unit, Nuffield Department of Women’s & Reproductive Health, University of Oxford, Oxford, UK

**Keywords:** EQ-5D-5L, Health-related quality of life, Degenerative lumbar spine, Spinal stenosis, Singapore, Patient-reported outcomes

## Abstract

**Objective:**

To examine sociodemographic, clinical, and healthcare-related factors associated with preoperative health-related quality of life (HRQoL) among patients undergoing surgery for degenerative lumbar spine conditions in a multi-ethnic Asian population.

**Methods:**

This cross-sectional study used baseline data from the Spine PROM Surgery Registry, including 1194 patients scheduled for surgery within a Singapore healthcare cluster between 2017 and 2022. HRQoL was measured using the EQ-5D-3L, with utility scores crosswalked to the EQ-5D-5L index using the van Hout crosswalk. Hierarchical linear regression assessed factors associated with HRQoL across three blocks: sociodemographic, clinical, and healthcare/lifestyle. Multivariable logistic regression identified factors associated with reporting problems within each EQ-5D dimension.

**Results:**

Mean age was 58.1 years (SD 16.1); 51.5% were male. Mean EQ-5D-5L crosswalk index was 0.43 (SD 0.38). Pain/discomfort (93.6%) and usual activities problems (84.3%) were most commonly reported. Lower EQ-5D scores were independently associated with non-outpatient presentation (β = −0.37), non-Chinese ethnicity, primary education or below relative to higher levels, and history of accident or trauma (β = −0.11). Dimension-level analyses showed primary education or below was associated with higher odds of problems across mobility, self-care, usual activities, and anxiety/depression compared with higher education levels, with a clear monotonic gradient. Female sex was associated with higher odds of pain/discomfort and anxiety/depression problems compared with male sex. Non-outpatient presentation was associated with markedly higher odds of self-care problems (OR = 2.98).

**Conclusions:**

Patients awaiting lumbar spine surgery appear to have impaired preoperative HRQoL. Although the modest explained variance limits robust risk prediction, preoperative profiles may still help inform clinical discussions and shared decision-making. Non-outpatient presentation may help identify patients who could benefit from enhanced preoperative support, although this requires prospective validation. Differences by ethnicity and education suggest opportunities for culturally tailored counselling. EQ-5D dimension profiles may indicate targets for prehabilitation and provide Singapore-based benchmark data for a lumbar spine surgery cohort for patient-centred care, service benchmarking, and health technology assessment.

**Supplementary Information:**

The online version contains supplementary material available at 10.1007/s11136-026-04257-1.

## Abstract (plain language summary)

Low back pain is a common reason why people lose the ability to work, move, and take part in daily life. Many patients with long-lasting back problems eventually need surgery, but their quality of life before surgery can vary widely. Understanding how patients feel and function before surgery is important for planning care, setting expectations, and deciding who may need extra support. This study looked at how good or poor quality of life is in patients waiting for surgery for degenerative lumbar spine conditions, and which personal, social, and clinical factors are linked to worse quality of life. We studied over 1100 patients in Singapore using a standard health questionnaire that measures mobility, self-care, daily activities, pain, and mental wellbeing. This allowed us to examine both overall quality of life and specific problem areas. We found that patients had substantial problems before surgery, especially with pain and daily activities. Quality of life did not differ by diagnosis, but was worse in patients who entered care through emergency or inpatient routes, and in some ethnic and educational groups. These findings suggest that identifying patients with poorer preoperative quality of life may help clinicians tailor support and plan care before surgery.

**Supplementary Information:**

The online version contains supplementary material available at 10.1007/s11136-026-04257-1.

## Introduction

Low back pain is the leading cause of disability worldwide, accounting for an estimated 70.4 million disability-adjusted life years globally in 2023 [1] and imposing a substantial clinical and economic burden, particularly in high-income countries [2]. Degenerative lumbar spine conditions, including spinal stenosis, disc herniation and spondylolisthesis, are common indications for surgery. Despite similar diagnostic labels, patients present with marked variation in preoperative health-related quality of life (HRQoL) impairment [3].

Systematic assessment of preoperative HRQoL has important prognostic and clinical value. Baseline HRQoL scores predict surgical outcomes, enabling clinicians to set realistic expectations and identify patients who may achieve greater postoperative benefit [4, 5]. In addition, EQ-5D dimension-level profiles provide insight into specific areas of impairment, such as mobility, self-care, or anxiety/depression, which can guide targeted counselling and preoperative intervention [6]. Identifying patients with particularly poor preoperative HRQoL may help clinicians recognise those who might benefit from additional preoperative assessment or support such as prehabilitation, pain management, and psychological support [4]. At a broader level, preoperative HRQoL data facilitate shared decision-making by providing benchmark data for similar surgical populations [5] and underpin health technology assessment through estimation of quality-adjusted life years gained from surgery [7, 8].

Based on findings from recent systematic reviews and an international panel of experts, the EQ-5D is recommended as the preferred instrument for measuring health-related quality of life in patients with low back pain [9, 10]. Previous studies have reported preoperative EQ-5D utility values ranging from 0.36 to 0.45 in patients with lumbar spine conditions [11], with pain or discomfort consistently identified as the most affected dimension [4, 12]. Several factors influence HRQoL in degenerative spine populations. Higher comorbidity burden is associated with poorer quality of life [13, 14], while obesity is linked to an increased low back pain prevalence and lower HRQoL [15, 16]. Depression shows a dose response relationship with chronic low back pain [17], and neuropathic pain components are associated with significantly reduced EQ-5D scores [18]. Socioeconomic factors, including educational attainment, independently influence disease burden and HRQoL outcomes [4, 19, 20]. Together, these findings suggest that preoperative HRQoL reflects both clinical severity and broader social determinants of health.

Nonetheless, most existing evidence derives from Western populations or relatively homogeneous East Asian cohorts [21]. Singapore represents a distinct context as a rapidly ageing, multi-ethnic Asian society comprising Chinese, Malay, Indian, and other ethnic groups. Although country-specific EQ-5D-3L value sets are available [22], describing preoperative HRQoL profiles and their associated factors in this setting remain limited. To our knowledge, this is the first study to provide a comprehensive, dimension-level characterisation of preoperative EQ-5D profiles and their sociodemographic, clinical, and healthcare-related correlates in a multi-ethnic Asian spine surgery cohort. This addresses an important gap, as most existing preoperative HRQoL data derive from Western or ethnically homogeneous populations, limiting their applicability to diverse Asian settings. Furthermore, the identification of modifiable and non-modifiable factors associated with preoperative HRQoL may inform the development of targeted prehabilitation strategies and culturally tailored care pathways. This study therefore aims to describe preoperative HRQoL using the EQ-5D-5L crosswalk index, identify factors associated with HRQoL, and examine dimension-specific associations to support patient counselling and prehabilitation.

## Methods

### Ethical approval

This study was performed in accordance with the Declaration of Helsinki. Ethical approval was granted by the Domain Specific Review Board, National Healthcare Group, Singapore (reference: 2025-1733). A waiver of informed consent was obtained as the study used anonymised registry data. This study was reported according to the Strengthening the Reporting of Observational Studies in Epidemiology (STROBE) guidelines [23].

### Study design, setting, and data source

This was a cross-sectional analysis of a single-centre spine surgery registry at a tertiary referral university spine centre comprising eight fellowship-trained spine surgeons. The Spine Surgery Registry was established in 2007 and has since routinely collected patient-reported outcome and clinical data from patients undergoing spine surgery. For the present study, the registry dataset comprised 1704 patients with lumbar spine conditions enrolled between 2017 and 2022. The registry prospectively enrolled adults with degenerative lumbar spine conditions who subsequently underwent lumbar surgery as part of routine care [4, 5]. PROMs were administered at surgery enlistment and during follow-up at 3–6 months, 1 year, and 2 years after surgery [4]. In this study, baseline referred to the preoperative time point at surgery enlistment, during the preoperative surgical consultation. A trained Spine Centre staff member collected PROM data using paper-based questionnaires during clinic visits, and the data were subsequently entered into Research Electronic Data Capture (REDCap) tools hosted locally within the hospital [24, 25]. PROM completion was near-universal because questionnaire administration was embedded in routine preoperative workflow; however, the registry did not maintain a formal response-rate denominator. Preoperative data collected between 2017 and 2022 were extracted for the present analysis.

### Eligibility criteria

Inclusion criteria were adults aged 18 years or older; a clinical diagnosis of lumbar spinal stenosis, lumbar disc herniation, lumbar spondylolisthesis, or degenerative disc disease; completion of preoperative EQ-5D-3L questionnaire; and availability of key sociodemographic and clinical variables. Exclusion criteria were: previous lumbar spine surgery; incomplete EQ-5D data; non-degenerative spinal pathology (e.g., tumour, infection, or trauma-related conditions); surgery cancellation; and surgery dates outside the study period.

### Study variables

Sociodemographic variables included age (further subcategorised into < 45, 45–64, and ≥ 65 years subgroups), sex, ethnicity, and educational attainment (primary or below, secondary, post-secondary/diploma, and university and above). Sex was defined as biological sex recorded in hospital administrative records. Clinical variables included body mass index (BMI; categorised using WHO Asia-Pacific cutoffs as normal/underweight [< 23 kg/m²], overweight [23–27.4 kg/m²], and obese [≥ 27.5 kg/m²]) [26, 27], comorbidity status (none versus ≥ 1 comorbidity), primary diagnosis (spinal stenosis, prolapsed disc, spondylolisthesis, or degenerative disc disease), and the spinal level(s) affected (L4/5, L4/5 and L5/S1, L5/S1, mixed level, or others). Healthcare and lifestyle factors included presentation pathway at registry recruitment (outpatient clinic vs. non-outpatient presentation via the emergency department or direct inpatient recruitment), a history of accident or trauma, and smoking status (never smoker, former smoker, or current smoker). Presentation pathway reflects the route of entry into spine care at registry recruitment and does not indicate surgical urgency.

### Patient-reported outcome measures

All patients completed the EQ-5D-3L questionnaire preoperatively, which describes health status across five dimensions (mobility, self-care, usual activities, pain/discomfort, and anxiety/depression) with three response levels each (no problems, some problems, and extreme problems) [6]. Index values were derived using the Singapore EQ-5D-3L value set [22], and responses were crosswalked to EQ-5D-5L values using the van Hout crosswalk to facilitate comparison with studies using the five-level instrument [28, 29].

### Statistical analysis

All analyses were conducted using Stata version 19.0 (StataCorp LLC, College Station, TX, USA) [30]. Continuous variables were summarised using means and standard deviations (SD); categorical variables were presented as counts and percentages. Differences across diagnostic groups were assessed using one-way analysis of variance (ANOVA) [31] for continuous variables and chi-square tests for categorical variables.

Hierarchical linear regression models were constructed to identify factors independently associated with HRQoL. The crosswalked EQ-5D-5L index served as the primary outcome, with the original EQ-5D-3L index examined in sensitivity analyses. Three sequential models were fitted: Model 1 included sociodemographic factors (age, sex, ethnicity, and education); Model 2 added clinical factors (BMI, comorbidity status, diagnosis, and lumbar spine level); and Model 3 further incorporated healthcare and lifestyle factors (presentation pathway, accident history, and smoking status). Model fit was evaluated using R², adjusted R², root mean square error (RMSE), Akaike information criterion (AIC), and Bayesian information criterion (BIC) [32].

Multivariable logistic regression models were fitted to examine factors associated with reporting any problems (level 2 or 3) versus no problems (level 1) for each EQ-5D-3L dimension separately. Odds ratios (OR) with 95% confidence intervals (CI) were reported. Model discrimination was assessed using the area under the receiver operating characteristic curve (AUC), and overall model fit was evaluated using pseudo R², AIC, and likelihood ratio chi-square statistics [33]. A two-sided p-value of < 0.05 was considered statistically significant. No adjustments for multiple comparisons were made given the exploratory nature of the analyses. All included patients had complete EQ-5D-3L responses, as complete EQ-5D data were an inclusion criterion. Missing data were minimal: spine level involvement had one missing observation (0.08%). Complete case analysis was used given the negligible proportion of missingness.

## Results

### Baseline characteristics

**Fig. 1:**
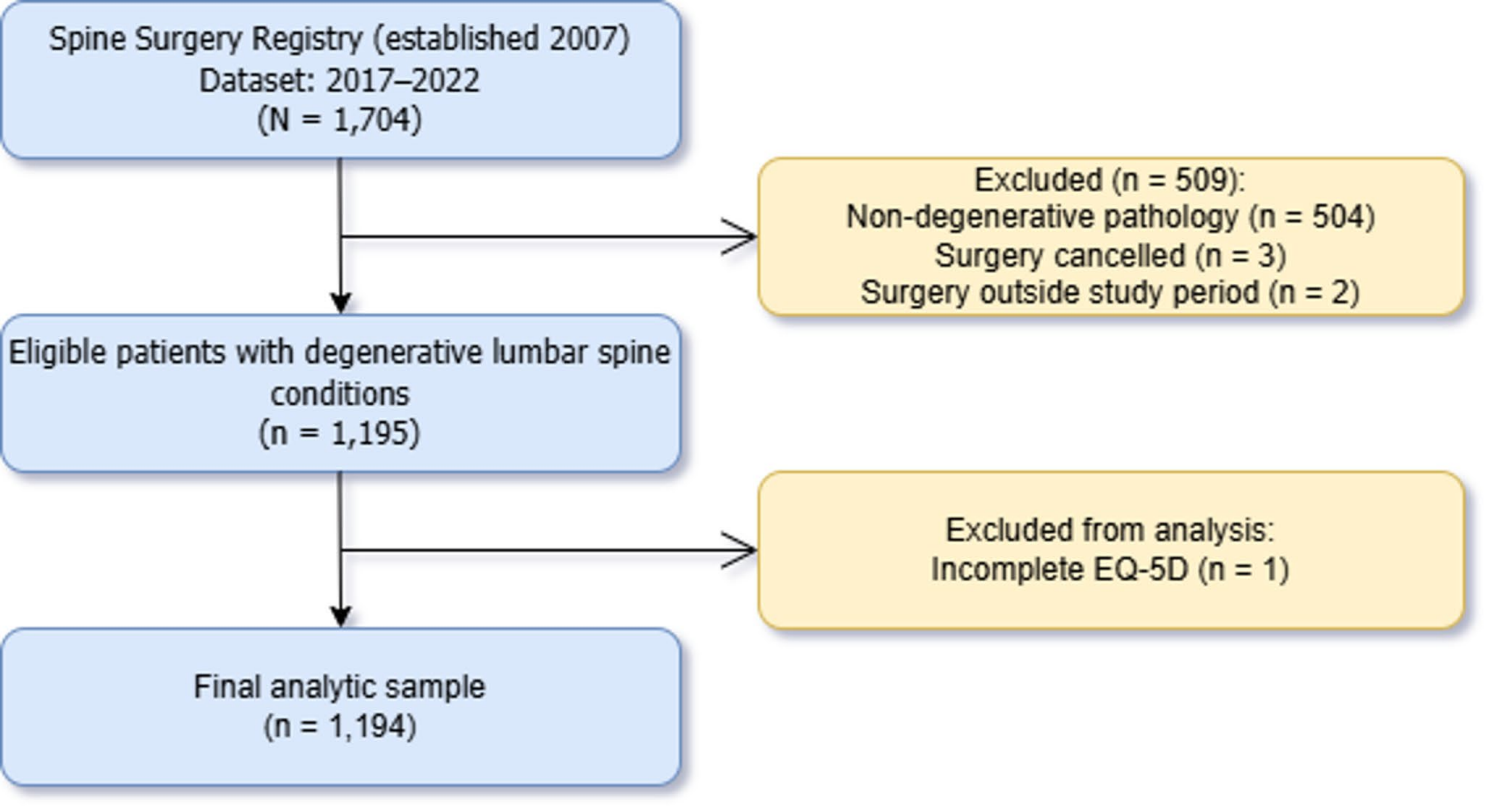
Patient flow diagram. Spine surgery registry, 2017–2022

Of the 1704 patients in the registry dataset, 504 were excluded for non-degenerative spinal pathology, 3 for surgery cancellation, 2 for surgery dates outside the study period, and 1 for incomplete EQ-5D-3L data, leaving 1194 patients who met all inclusion criteria. A patient flow diagram is presented in Fig. 1. The baseline characteristics of the study population are presented in Table 1. The overall mean age was 58.1 years (SD 16.1), with 51.5% of the cohort being male. The age distribution showed that 42.4% were older adults (≥ 65 years), 37.1% were middle-aged (45–64 years), and 20.5% were young adults (< 45 years). The majority were of Chinese ethnicity (70.3%), followed by Malay (12.1%), others (9.0%), and Indian (8.6%). Highest educational level attainment was distributed across the various categories as follows: primary or below (19.9%), secondary (31.5%), post-secondary/diploma (25.8%), and university and above (22.9%).

**Table 1:** Baseline characteristics and health-related quality of life of patients with degenerative lumbar spine conditions

Characteristic	*n* (%) or Mean (SD)
*Socio-demographic characteristics*	
Age (years), mean (SD)	58.1 (16.1)
Age category	
Young adults (< 45 years)	245 (20.5)
Middle-aged (45–64 years)	443 (37.1)
Older adults (≥ 65 years)	506 (42.4)
Sex	
Male	615 (51.5)
Female	579 (48.5)
Race/ethnicity	
Chinese	839 (70.3)
Malay	145 (12.1)
Indian	103 (8.6)
Others	107 (9.0)
Education level	
Primary or below	237 (19.9)
Secondary	376 (31.5)
Post-secondary/Diploma	308 (25.8)
University and above	273 (22.9)
*Clinical characteristics*	
BMI (kg/m²), mean (SD)	26.3 (4.7)
BMI category (Asian cutoffs)	
Normal/Underweight (< 23)	289 (24.2)
Overweight (23–27.4)	492 (41.2)
Obese (≥ 27.5)	413 (34.6)
Comorbidity status	
No comorbidities	377 (31.6)
≥ 1 comorbidity	817 (68.4)
Diagnosis	
Spinal stenosis	483 (40.5)
Prolapsed intervertebral disc	273 (22.9)
Spondylolisthesis	236 (19.8)
Degenerative disc disease	202 (16.9)
Spine level involvement	
L4/5	437 (36.6)
L4/5 and L5/S1	128 (10.7)
L5/S1	202 (16.9)
Mixed level	82 (6.9)
Others	344 (28.8)
*Healthcare and lifestyle factors*	
Presentation pathway	
Outpatient clinic	1118 (93.6)
Non-outpatient presentation	76 (6.4)
History of accident/trauma	
No	1131 (94.7)
Yes	63 (5.3)
Smoking history	
Never smoker	933 (78.1)
Former smoker	102 (8.5)
Current smoker	159 (13.3)
*Health-related quality of life*	
EQ-5D-5L crosswalk index, mean (SD)	0.43 (0.38)
EQ-5D-3L index, mean (SD)	0.48 (0.34)
EQ-5D-3L dimensions with any problems	
Mobility	885 (74.1)
Self-care	457 (38.3)
Usual activities	1007 (84.3)
Pain/discomfort	1118 (93.6)
Anxiety/depression	634 (53.1)

BMI, body mass index; EQ-5D-3L, 3-level EQ-5D; EQ-5D-5L, 5-level EQ-5D; SD, standard deviation

Data are presented as n (%) unless otherwise indicated. BMI categories based on WHO Asia-Pacific cutoffs. EQ-5D-5L crosswalk index derived using crosswalk algorithm from EQ-5D-3L responses. Presentation pathway at registry recruitment indicates the route by which patients entered the spine care pathway at recruitment: outpatient clinic vs. non-outpatient presentation (emergency department or direct inpatient recruitment). ‘Any problems’ includes levels 2–3 (some or extreme problems). Missing data: spine level (*n* = 1)

The mean BMI was 26.3 kg/m² (SD 4.7), with 34.6% classified as obese, 41.2% as overweight, and 24.2% as normal or underweight. Most patients (68.4%) had at least one comorbidity. The most common diagnosis was spinal stenosis (40.5%), followed by a prolapsed intervertebral disc (22.9%), spondylolisthesis (19.8%), and degenerative disc disease (16.9%). The most frequently affected lumbar spine level was L4/5 (36.6%). Most patients (93.6%) entered care via outpatient clinics, 5.3% had a history of accident or trauma, and 78.1% reported having never smoked.

### Health-related quality of life outcomes

**Fig. 2:**
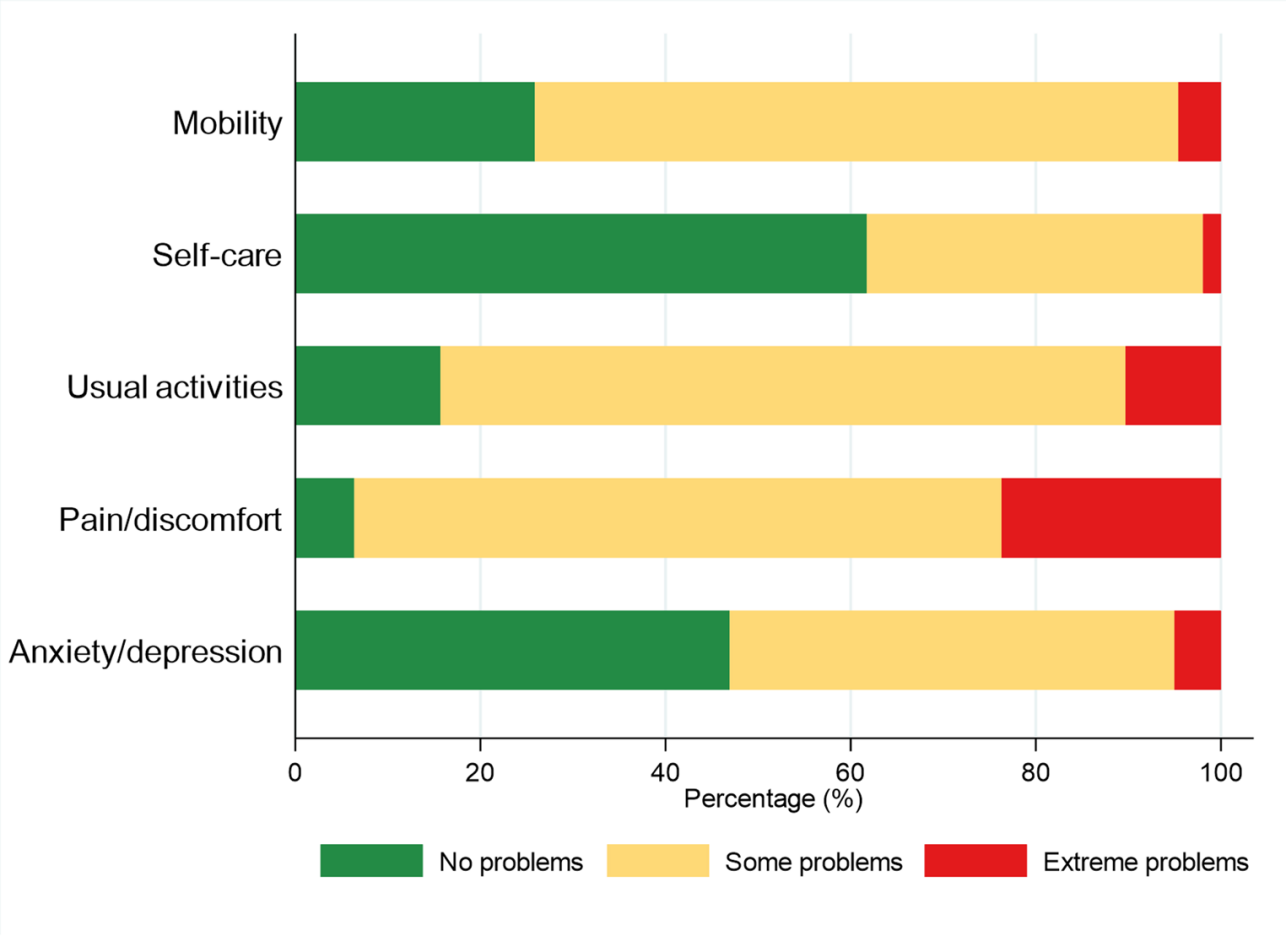
Distribution of EQ-5D health dimensions by severity level

The overall mean EQ-5D-3L index was 0.48 (SD 0.34) and the crosswalked EQ-5D-5L index was 0.43 (SD 0.38) (Table 1). Analysis of EQ-5D-3L dimension responses revealed that pain/discomfort was the most affected dimension, with 93.6% of patients reporting some or extreme problems (Fig. 2). Usual activities were also highly affected (84.3% with problems), followed by mobility (74.1%), anxiety/depression (53.1%), and self-care (38.3%).

### Univariate associations with health-related quality of life

Table 2 presents the univariate associations between independent variables and HRQoL outcomes. Sex was significantly associated with EQ-5D-5L index (*p* = 0.003), with males reporting higher mean scores than females (0.46 versus 0.39). Ethnicity showed significant differences (*p* < 0.001), with Chinese patients reporting the highest mean EQ-5D-5L index (0.46) compared with Malay (0.32), Indian (0.34), and other ethnicities (0.37). Educational attainment differed significantly across categories, with university-educated patients reporting the highest scores (0.48) and those with primary education or below the lowest (0.32).

**Table 2:** Univariate analyses of health-related quality of life by predictor categories

Characteristic	*n*	EQ-5D-5L crosswalk index	*p*
*Socio-demographic*			
Age category			0.851
Young adults (< 45)	245	0.41 (0.42)	
Middle-aged (45–64)	443	0.43 (0.38)	
Older adults (≥ 65)	506	0.43 (0.36)	
Sex			0.003
Male	615	0.46 (0.38)	
Female	579	0.39 (0.38)	
Race/ethnicity			< 0.001
Chinese	839	0.46 (0.36)	
Malay	145	0.32 (0.44)	
Indian	103	0.34 (0.41)	
Others	107	0.37 (0.42)	
Education level			< 0.001
Primary or below	237	0.32 (0.39)	
Secondary	376	0.42 (0.37)	
Post-secondary/Diploma	308	0.46 (0.36)	
University and above	273	0.48 (0.39)	
*Clinical*			
BMI category			0.002
Normal/Underweight (< 23)	289	0.43 (0.41)	
Overweight (23–27.4)	492	0.46 (0.35)	
Obese (≥ 27.5)	413	0.38 (0.39)	
Comorbidity status			0.360
No comorbidities	377	0.44 (0.40)	
≥ 1 comorbidity	817	0.42 (0.37)	
Diagnosis			0.526
Spinal stenosis	483	0.43 (0.37)	
Prolapsed intervertebral disc	273	0.40 (0.42)	
Spondylolisthesis	236	0.45 (0.35)	
DDD	202	0.41 (0.38)	
Spine level involvement			0.180
L4/5	437	0.44 (0.38)	
L4/5 and L5/S1	128	0.46 (0.35)	
L5/S1	202	0.40 (0.42)	
Mixed level	82	0.35 (0.38)	
Others	344	0.43 (0.37)	
*Healthcare and lifestyle*			
Presentation pathway			< 0.001
Outpatient clinic	1118	0.45 (0.35)	
Non-outpatient presentation	76	0.04 (0.56)	
History of accident/trauma			< 0.001
No	1131	0.43 (0.37)	
Yes	63	0.26 (0.46)	
Smoking history			0.071
Never smoker	933	0.44 (0.37)	
Former smoker	102	0.41 (0.38)	
Current smoker	159	0.36 (0.42)	

BMI, body mass index; DDD, degenerative disc disease

Data are presented as mean (SD). EQ-5D-5L crosswalk index derived using crosswalk algorithm. Presentation pathway at registry recruitment indicates the route by which patients entered the spine care pathway at recruitment: outpatient clinic vs. non-outpatient presentation (emergency department or direct inpatient recruitment)

*p*-values from independent samples t-test (binary variables) or one-way ANOVA (categorical variables with > 2 groups)

BMI was also significantly associated with the EQ-5D-5L index (*p* = 0.002), with obese patients reporting lower scores. Presentation pathway showed the largest univariate differences: non-outpatient presentation, reflecting the route of presentation into care at registry recruitment, was associated with substantially lower EQ-5D-5L index (0.04 versus 0.45, *p* < 0.001). A history of accident or trauma was also significantly associated with poorer HRQoL (*p* < 0.001 for EQ-5D-5L index). Notably, there were no statistically significant differences in HRQoL outcomes across the different diagnosis groups (*p* = 0.526 for EQ-5D-5L index).

### Hierarchical regression analyses for EQ-5D-5L index

Table 3 presents the hierarchical linear regression models for the crosswalked EQ-5D-5L index. In Model 1 (sociodemographic factors only), male sex was associated with higher EQ-5D-5L index (β = 0.05, 95% CI: 0.01 to 0.09, *p* < 0.05). Compared with primary education or below, higher educational attainment was associated with progressively higher EQ-5D-5L index, in a monotonic gradient: secondary (β = 0.11, 95% CI: 0.05 to 0.17, *p* < 0.001), post-secondary or Diploma (β = 0.17, 95% CI: 0.10 to 0.23, *p* < 0.001), and university and above (β = 0.19, 95% CI: 0.12 to 0.26, *p* < 0.001). Model 1 explained 5.7% of the variance in EQ-5D-5L index.

**Table 3:** Hierarchical linear regression of factors associated with EQ-5D-5L crosswalk index

Characteristic	Model 1	Model 2	Model 3
	β (95% CI)	β (95% CI)	β (95% CI)
*Socio-demographic*			
Age category			
Young adults (< 45)	Ref	Ref	Ref
Middle-aged (45–64)	0.03 (−0.03, 0.09)	0.01 (−0.06, 0.08)	−0.01 (−0.07, 0.06)
Older adults (≥ 65)	0.03 (−0.03, 0.09)	0.00 (−0.08, 0.08)	−0.02 (−0.09, 0.06)
Sex			
Male	Ref	Ref	Ref
Female	−0.05 (−0.09, −0.01)*	−0.05 (−0.09, −0.00) *	−0.06 (−0.10, −0.01)*
Race/ethnicity			
Chinese	Ref	Ref	Ref
Malay	−0.14 (−0.21, −0.07) ***	−0.13 (−0.20, −0.06) ***	−0.08 (−0.15, −0.01) *
Indian	−0.14 (−0.22, −0.06) ***	−0.14 (−0.21, −0.06)***	−0.10 (−0.17,−0.02) *
Others	−0.14 (−0.22, −0.06) ***	−0.13 (− 0.21, − 0.06) ***	−0.12 (− 0.20, − 0.05) **
Education level			
Primary or below	Ref	Ref	Ref
Secondary	0.11 (0.05, 0.17) ***	0.11 (0.05, 0.17) ***	0.10 (0.04, 0.16) ***
Post-secondary/Diploma	0.17 (0.10, 0.23)***	0.16 (0.10, 0.23)***	0.15 (0.09, 0.21) ***
University and above	0.19 (0.12, 0.26)***	0.19 (0.12, 0.26) ***	0.16 (0.09, 0.23) ***
*Clinical*			
BMI category			
Normal/Underweight (< 23)	—	Ref	Ref
Overweight (23–27.4)	—	0.03 (− 0.03, 0.08)	0.03 (− 0.03, 0.08)
Obese (≥ 27.5)	—	−0.03 (− 0.09, 0.02)	−0.03 (− 0.09, 0.02)
Comorbidity status			
No comorbidities	—	Ref	Ref
≥ 1 comorbidity	—	−0.03 (− 0.08, 0.03)	−0.03 (− 0.08, 0.02)
Diagnosis			
Spinal stenosis	—	Ref	Ref
Prolapsed intervertebral disc	—	−0.05 (− 0.12, 0.01)	−0.03 (− 0.10, 0.04)
Spondylolisthesis	—	0.01 (− 0.05, 0.07)	0.00 (− 0.06, 0.06)
DDD	—	−0.03 (− 0.09, 0.03)	−0.03 (− 0.09, 0.03)
Spine level involvement			
L4/5	—	Ref	Ref
L4/5 and L5/S1	—	0.02 (− 0.06, 0.09)	0.04 (− 0.03, 0.11)
L5/S1	—	−0.05 (− 0.11, 0.02)	−0.04 (− 0.10, 0.03)
Mixed level	—	−0.07 (− 0.16, 0.02)	−0.05 (− 0.13, 0.04)
Others	—	−0.01 (− 0.06, 0.05)	0.00 (− 0.05, 0.06)
*Healthcare and lifestyle*			
Presentation pathway			
Outpatient clinic	—	—	Ref
Non-outpatient presentation	—	—	−0.37 (− 0.46, − 0.28) ***
History of accident/trauma			
No	—	—	Ref
Yes	—	—	−0.11 (− 0.20, − 0.01) *
Smoking history			
Never smoker	—	—	Ref
Former smoker	—	—	−0.05 (− 0.13, 0.03)
Current smoker	—	—	−0.07 (− 0.13, − 0.00)*
*Model fit indices*			
R²	0.057	0.069	0.132
Adjusted R²	0.049	0.054	0.115
AIC	1036.4	1040.4	964.8
BIC	1087.3	1142.1	1086.8
F statistic	7.88***	4.58***	7.74***
ΔR²	—	0.012	0.063

β, unstandardized regression coefficient; AIC, Akaike information criterion; BIC, Bayesian information criterion; BMI, body mass index; CI, confidence interval; DDD, degenerative disc disease

Model 1: Socio-demographic factors; Model 2: Model 1 + clinical factors; Model 3: Model 2 + healthcare and lifestyle factors. EQ-5D-5L crosswalk index derived using crosswalk algorithm. Presentation pathway at registry recruitment indicates the route by which patients entered the spine care pathway at recruitment: outpatient clinic vs. non-outpatient presentation (emergency department or direct inpatient recruitment)

ΔR² represents change in R² from previous model. *p < 0.05; **p < 0.01; ***p < 0.001

The addition of clinical factors in Model 2 did not substantially improve model fit (ΔR²=0.012), and no clinical variables achieved statistical significance. In the full model (Model 3), non-outpatient presentation showed the largest coefficient magnitude associated with lower HRQoL scores (β=−0.37, 95% CI: −0.46 to −0.28, *p* < 0.001). Primary education or below, non-Chinese ethnicity and history of accident or trauma remained independently associated with poorer HRQoL. The full model explained 13.2% of the variance in EQ-5D-5L index (adjusted R²=0.115).

### Factors associated with problems in individual EQ-5D-3L dimensions

Table 4 presents the multivariable logistic regression results for reporting any problems in each EQ-5D-3L dimension. The patterns of association varied across dimensions.

For mobility, higher education was associated with progressively lower odds of reporting problems compared with primary education or below, in a clear monotonic gradient: secondary (OR = 0.53, 95% CI: 0.33 to 0.85, *p* < 0.01), post-secondary or Diploma (OR = 0.37, 95% CI: 0.23 to 0.60, *p* < 0.001), and university and above (OR = 0.25, 95% CI: 0.15 to 0.41, *p* < 0.001). The model demonstrated acceptable discrimination (AUC = 0.68) (Supplementary Table 1).

**Table 4:** Multivariable logistic regression of predictors of reporting any problems in EQ-5D-3L dimensions

Characteristic	Mobility	Self-care	Usual activities	Pain/discomfort	Anxiety/depression
	OR (95% CI)	OR (95% CI)	OR (95% CI)	OR (95% CI)	OR (95% CI)
*Socio-demographic*					
Age category					
Young adults (< 45)	Ref	Ref	Ref	Ref	Ref
Middle-aged (45–64)	1.15 (0.76, 1.74)	1.02 (0.68, 1.51)	0.68 (0.40, 1.16)	0.48 (0.19, 1.16)	1.07 (0.74, 1.56)
Older adults (≥ 65)	1.41 (0.86, 2.32)	1.27 (0.80, 2.01)	0.57 (0.31, 1.07)	0.30 (0.11, 0.81) *	0.80 (0.51, 1.24)
Sex					
Male	Ref	Ref	Ref	Ref	Ref
Female	1.33 (0.99, 1.79)	1.29 (0.99, 1.69)	1.26 (0.89, 1.81)	1.90 (1.12, 3.22)*	1.40 (1.08, 1.81) **
Race/ethnicity					
Chinese	Ref	Ref	Ref	Ref	Ref
Malay	1.27 (0.79, 2.04)	1.20 (0.80, 1.78)	1.15 (0.64, 2.06)	3.14 (0.92, 10.66)	1.04 (0.70, 1.53)
Indian	1.76 (1.03, 3.02)*	1.17 (0.75, 1.83)	1.37 (0.73, 2.59)	1.28 (0.51, 3.20)	1.08 (0.70, 1.67)
Others	1.28 (0.80, 2.07)	1.38 (0.89, 2.16)	3.40 (1.57, 7.37) **	2.23 (0.77, 6.52)	1.07 (0.70, 1.64)
Education level					
Primary or below	Ref	Ref	Ref	Ref	Ref
Secondary	0.53 (0.33, 0.85) **	0.61 (0.43, 0.86) **	0.60 (0.35, 1.03)	0.80 (0.38, 1.68)	0.65 (0.46, 0.92) *
Post-secondary/Diploma	0.37 (0.23, 0.60) ***	0.53 (0.37, 0.78) **	0.55 (0.31, 0.98) *	0.57 (0.26, 1.23)	0.51 (0.35, 0.74) ***
University and above	0.25 (0.15, 0.41) ***	0.41 (0.27, 0.62) ***	0.32 (0.18, 0.57) ***	0.52 (0.23, 1.18)	0.44 (0.29, 0.65) ***
*Clinical*					
BMI category					
Normal/Underweight (< 23)	Ref	Ref	Ref	Ref	Ref
Overweight (23–27.4)	1.11 (0.78, 1.56)	1.13 (0.82, 1.56)	0.82 (0.54, 1.25)	1.44 (0.79, 2.60)	0.87 (0.64, 1.18)
Obese (≥ 27.5)	1.41 (0.97, 2.05)	1.49 (1.07, 2.08) *	1.15 (0.72, 1.82)	1.15 (0.61, 2.16)	0.89 (0.64, 1.22)
Comorbidity status					
No comorbidities	Ref	Ref	Ref	Ref	Ref
≥ 1 comorbidity	1.21 (0.87, 1.69)	1.09 (0.80, 1.48)	1.89 (1.28, 2.80) **	1.55 (0.87, 2.77)	1.24 (0.92, 1.66)
Diagnosis					
Spinal stenosis	Ref	Ref	Ref	Ref	Ref
Prolapsed intervertebral disc	1.11 (0.72, 1.69)	0.92 (0.62, 1.37)	0.80 (0.48, 1.34)	1.22 (0.55, 2.67)	1.31 (0.90, 1.91)
Spondylolisthesis	0.98 (0.67, 1.43)	0.80 (0.57, 1.12)	0.82 (0.53, 1.27)	1.28 (0.65, 2.51)	1.35 (0.98, 1.87)
DDD	1.97 (1.26, 3.08) **	0.95 (0.67, 1.36)	1.89 (1.08, 3.32) *	1.24 (0.62, 2.49)	1.16 (0.82, 1.65)
Spine level involvement					
L4/5	Ref	Ref	Ref	Ref	Ref
L4/5 and L5/S1	0.98 (0.61, 1.58)	0.74 (0.48, 1.15)	0.81 (0.46, 1.43)	1.87 (0.70, 5.02)	1.25 (0.83, 1.89)
L5/S1	0.89 (0.60, 1.33)	1.19 (0.82, 1.74)	1.27 (0.75, 2.15)	1.28 (0.56, 2.93)	1.25 (0.87, 1.80)
Mixed level	1.51 (0.78, 2.92)	1.13 (0.68, 1.87)	1.28 (0.59, 2.78)	1.06 (0.41, 2.76)	1.60 (0.96, 2.64)
Others	1.04 (0.73, 1.50)	0.85 (0.62, 1.17)	0.80 (0.52, 1.21)	1.03 (0.57, 1.87)	1.07 (0.79, 1.45)
*Healthcare and lifestyle*					
Presentation pathway					
Outpatient clinic	Ref	Ref	Ref	Ref	Ref
Non-outpatient presentation	1.50 (0.79, 2.82)	2.98 (1.79, 4.97) ***	2.03 (0.84, 4.92)	0.45 (0.19, 1.07)	1.57 (0.94, 2.60)
History of accident/trauma					
No	Ref	Ref	Ref	Ref	Ref
Yes	1.82 (0.88, 3.76)	1.20 (0.69, 2.08)	5.14 (1.22, 21.62) *	1.02 (0.30, 3.48)	0.96 (0.56, 1.65)
Smoking history					
Never smoker	Ref	Ref	Ref	Ref	Ref
Former smoker	1.39 (0.83, 2.34)	1.16 (0.74, 1.84)	1.77 (0.91, 3.47)	2.47 (0.85, 7.18)	1.28 (0.83, 1.99)
Current smoker	1.17 (0.75, 1.80)	1.29 (0.88, 1.88)	1.23 (0.70, 2.14)	1.26 (0.56, 2.87)	1.01 (0.70, 1.47)
Model fit indices					
Pseudo R²	0.071	0.046	0.074	0.061	0.032
AUC	0.68	0.64	0.70	0.70	0.62
AIC	1313.7	1563.3	1004.1	579.1	1645.1
LR χ²	97.04***	72.66***	76.62***	34.52	52.07***

AIC, Akaike information criterion; AUC, area under the receiver operating characteristic curve; BMI, body mass index; CI, confidence interval; DDD, degenerative disc disease; LR, likelihood ratio; OR, odds ratio

‘Any problems’ defined as reporting level 2 or 3 (some problems or extreme problems) vs. level 1 (no problems). Models adjusted for all variables shown. Presentation pathway at registry recruitment indicates the route by which patients entered the spine care pathway at recruitment: outpatient clinic vs. non-outpatient presentation (emergency department or direct inpatient recruitment)

*p < 0.05; **p < 0.01; ***p < 0.001

For self-care, non-outpatient presentation showed the largest odds ratio for reporting problems (OR = 2.98, 95% CI: 1.79 to 4.97, *p* < 0.001). Obesity was associated with increased odds of self-care problems (OR = 1.49, 95% CI: 1.07 to 2.08, *p* < 0.05). Attaining higher education was also associated with decreased odds compared with primary education or below (OR = 0.61, 95% CI: 0.43 to 0.86, *p* < 0.01). Model discrimination was modest (AUC = 0.64).

For usual activities, having one or more comorbidities nearly doubled the odds of reporting problems (OR = 1.89, 95% CI: 1.28 to 2.80, *p* < 0.01). A history of accident or trauma was strongly associated with increased odds (OR = 5.14, 95% CI: 1.22 to 21.62, *p* < 0.05). Patients of other ethnicity had higher odds compared with Chinese patients (OR = 3.40, 95% CI: 1.57 to 7.37, *p* < 0.01). Model discrimination was acceptable (AUC = 0.70).

For pain/discomfort, females had higher odds of reporting problems compared with males (OR = 1.90, 95% CI: 1.12 to 3.22, *p* < 0.05). Model discrimination was acceptable (AUC = 0.70).

For anxiety/depression, female sex was associated with increased odds of reporting problems (OR = 1.40, 95% CI: 1.08 to 1.81, *p* < 0.01). Higher education was associated with progressively reduced odds of anxiety/depression problems compared with primary education or below: secondary (OR = 0.65, 95% CI: 0.46 to 0.92, *p* < 0.05), post-secondary or Diploma (OR = 0.51, 95% CI: 0.35 to 0.74, *p* < 0.001), and university and above (OR = 0.44, 95% CI: 0.29 to 0.65, *p* < 0.001). Model discrimination was modest (AUC = 0.62).

### Sensitivity analyses

Supplementary Table 2 presents sensitivity analyses using the original EQ-5D-3L index. The direction and statistical significance of associations were consistent with those observed for the crosswalked EQ-5D-5L index. Non-outpatient presentation remained the variable with the largest coefficient magnitude for lower HRQoL (β = −0.33, 95% CI: −0.41 to −0.26, *p* < 0.001), and the full model explained 12.8% of the variance. Supplementary Table 3 provides a direct comparison of the full model results for both indices, demonstrating robust consistency across the two outcome measures.

## Discussion

This study characterised preoperative HRQoL in a large, multi-ethnic cohort of patients with degenerative lumbar spine conditions at a tertiary spine centre in Singapore. The principal findings were: (1) substantial HRQoL impairment, with mean EQ-5D-5L crosswalk index of 0.43; (2) pain/discomfort and usual activities were the most affected dimensions; (3) no significant HRQoL differences across diagnostic groups; (4) non-outpatient presentation at registry recruitment emerged as the variable with the largest coefficient magnitude associated with poorer HRQoL, exceeding established minimal clinically important differences; and (5) significant associations between ethnicity, education, and HRQoL persisted after multivariable adjustment. Importantly, the full model explained only 13.2% of the variance in EQ-5D-5L index (adjusted R² = 0.115), indicating that unmeasured factors contribute substantially to preoperative HRQoL variation. The identified associations should therefore be interpreted as exploratory rather than as a basis for definitive risk prediction.

### Comparison with existing literature

Our mean EQ-5D-5L crosswalk index of 0.43 and EQ-5D-3L index of 0.48 align with pooled estimates from meta-analyses reporting preoperative utilities of 0.29–0.46 for lumbar spine conditions [3, 11]. The dimensional profile, with pain/discomfort most affected (93.6%), followed by usual activities (84.3%), mirrors global patterns [4, 12]. This near-universal prevalence of pain-related problems reflects the symptomatic threshold required for surgical consideration and underscores that pain management remains a central therapeutic target. The absence of significant HRQoL differences across diagnostic groups contrasts with some studies [3], but aligns with evidence that patient-level characteristics contribute more to HRQoL variation than disease-specific factors [4, 21], supporting standardised preoperative assessment protocols across degenerative lumbar conditions without requiring diagnosis-specific modifications.

### Association between non-outpatient presentation and baseline HRQoL

Non-outpatient presentation emerged as the variable showing the largest coefficient magnitude for lower HRQoL (β = −0.37), substantially exceeding established minimal clinically important differences [34]. Importantly, this variable reflects the route of presentation into the spine care pathway at registry recruitment rather than admission status at the point of surgery. It therefore distinguishes patients presenting via the emergency department or direct inpatient recruitment from those referred through outpatient clinics, without implying surgical urgency. The association between non-outpatient presentation and poorer baseline HRQoL may reflect underlying patient characteristics, such as more severe or disabling symptoms at presentation. The cross-sectional design precludes causal inference, and the association between non-outpatient presentation and poorer HRQoL may be substantially confounded by unmeasured disease severity, acuity of symptoms, or healthcare access patterns. Rather than representing an independent actionable factor, non-outpatient presentation may serve as a proxy marker for patients with more severe or disabling conditions. Nonetheless, patients presenting via non-outpatient pathways could be considered for enhanced preoperative assessment, pending prospective validation. This may include enhanced functional assessment, realistic expectation-setting, and closer postoperative monitoring [35]. Clinicians may therefore consider flagging non-outpatient presentations for enhanced preoperative care pathways.

### Addressing sociodemographic disparities

Non-Chinese ethnicities (Malay, Indian, and others) reported significantly lower EQ-5D indices even after adjustment for education, BMI, comorbidity, and clinical factors. Similar ethnic variations have been documented in previous reviews [36]. Several mechanisms may underlie these disparities. These include potential differences in pain perception, illness behaviour, health beliefs, or unmeasured socioeconomic confounders such as household income, occupational exposures, and insurance status, which were not captured in the registry. The observed associations should be interpreted with caution, as residual confounding by these unmeasured factors cannot be excluded [19, 21]. These findings highlight opportunities for culturally sensitive practice. Patients from minority ethnic groups may benefit from culturally tailored education and counselling, including attention to health beliefs and potential barriers to postoperative rehabilitation [37]. These disparities also highlight potential limitations in applying uniform value sets across diverse populations.

A clear monotonic gradient was observed between higher education and better preoperative HRQoL. Compared with primary education or below, all higher education categories were associated with lower odds of problems across mobility, self-care, usual activities, and anxiety/depression, with the strongest effects in the university-educated group. This pattern is consistent with a substantial body of evidence linking higher educational attainment to better health literacy, more effective health-seeking behaviour, and lower disease burden in chronic conditions [19, 20]. Patients with primary education or below also had higher odds of anxiety or depression problems, suggesting a potential role for psychological screening and culturally adapted patient education in this subgroup. Clinicians should consider the role of educational background in shaping patient expectations and engagement with preoperative care.

Female sex was associated with higher odds of reporting pain/discomfort and anxiety/depression problems and with marginally lower preoperative EQ-5D index scores. These findings are consistent with prior literature documenting that women undergoing lumbar spine surgery often present with greater preoperative pain and psychological distress [38, 39, 40, 41]. Possible explanations include sex-based differences in pain perception and reporting [21], cumulative musculoskeletal burden related to occupational and caregiving roles, and culturally shaped help-seeking patterns where women may delay surgical consultation until symptoms become more severe, with evidence that help-seeking tendencies differ between Western and Asian populations [42]. These findings suggest that female patients awaiting lumbar spine surgery may benefit from targeted preoperative pain management and psychological support, and that referral pathways should be examined to ensure timely access for women presenting with degenerative lumbar conditions.

### Dimension-specific factors

The logistic regression analyses revealed distinct patterns across EQ-5D dimensions, reinforcing the value of dimension-level assessment alongside summary indices. Degenerative disc disease was associated with higher odds of mobility problems compared with spinal stenosis (OR = 1.97). This may reflect differences in clinical presentation, as degenerative disc disease often affects younger patients with discogenic pain exacerbated by axial loading [3], whereas spinal stenosis typically involves gradual progression and neurogenic claudication [43]. These findings support condition-specific counselling regarding expected functional limitations.

Comorbidity burden was associated with nearly doubled odds of problems with usual activities (OR = 1.89), consistent with evidence that multimorbidity imposes cumulative functional burden [14, 18]. The specific association with usual activities, rather than mobility or self-care, suggests that functional impact may relate more to reduced stamina and energy expenditure than to mechanical limitations. Patients with multiple comorbidities may therefore benefit from comprehensive preoperative assessment to anticipate rehabilitation needs and coordinate postoperative care.

Older adults demonstrated lower odds of reporting pain or discomfort problems (OR = 0.30). This finding may reflect age-related changes in pain processing, adapted expectations, or generational differences in symptom reporting [21]. Clinicians should supplement standardised patient-reported outcome measures with direct functional assessment, recognising that older patients may underreport pain. Obesity was associated with higher odds of self-care problems (OR = 1.49), which may reflect biomechanical challenges related to personal care tasks [15, 16]. Rather than advocating unrealistic preoperative weight loss, clinicians should prioritise expectation-setting and ensure adequate home and caregiver support. The modest discrimination observed across models (AUC 0.62–0.70) indicates limited clinical applicability for individual-level prediction and suggests that unmeasured factors contribute substantially to dimension-specific patterns. These AUC values fall below thresholds typically considered adequate for clinical decision tools (AUC ≥ 0.75), and the findings should therefore be interpreted as descriptive and hypothesis-generating rather than as a basis for clinical prediction. Prospective validation in independent cohorts would be necessary before any clinical application.

### Strengths and limitations

This study has several strengths. The large, multi-ethnic cohort enabled examination of sociodemographic disparities that are rarely captured in more homogeneous populations. The use of locally validated Singapore EQ-5D-3L value sets supports culturally appropriate utility estimation [22]. The hierarchical regression approach allowed systematic evaluation of the contribution of individual variables, while sensitivity analyses demonstrated consistent findings across both EQ-5D indices. The registry-based design enhances ecological validity.

Several limitations merit consideration, alongside the steps taken to address them. First, the cross-sectional design precludes causal inference; a companion study examining postoperative change scores from the same registry has been published to address this [4]. Second, the registry did not capture symptom duration, neuropathic pain components, psychological distress, or detailed socioeconomic indicators; we have discussed these as potential unmeasured confounders and recommended their inclusion in future studies. Third, smoking history was self-reported and may be subject to misclassification, although observed distributions were consistent with population-level data. Fourth, the absence of disease-specific measures such as the Oswestry Disability Index limits comparisons with some studies; however, the EQ-5D is the recommended generic preference-based measure for low back pain populations [9, 10] and enables health economic evaluation. Fifth, reliance on crosswalk transformation may have reduced measurement precision; to address this, sensitivity analyses using the original EQ-5D-3L index were conducted and demonstrated consistent findings (Supplementary Tables 2 and 3) [29]. Sixth, the single-centre tertiary setting may limit generalisability to primary care or community hospitals; multi-centre replication across Singapore’s healthcare clusters would strengthen external validity. Seventh, comorbidity was assessed using an overall burden measure rather than individual conditions, as small sample sizes for some comorbidities precluded robust modelling; this may have obscured condition-specific effects on HRQoL, and future registries should consider capturing individual comorbidity data. Eighth, normal and underweight BMI categories were combined due to small sample sizes, which may limit the generalisability of BMI-related findings to these subgroups and warrants cautious interpretation. Ninth, the wide confidence interval for accident or trauma history (OR = 5.14, 95% CI: 1.22 to 21.62) reflects the small number of affected patients and indicates substantial imprecision, warranting cautious interpretation. Tenth, no adjustments for multiple comparisons were applied, particularly in the five dimension-level logistic regression models; given the exploratory nature of these analyses, the risk of type I error should be considered and findings from dimension-level models should be regarded as hypothesis-generating. Finally, the modest proportion of variance explained (11.5%) indicates that unmeasured factors substantially influence preoperative HRQoL; the hierarchical regression approach was used to systematically assess the incremental contribution of each variable block, and future research should incorporate additional predictors such as symptom duration, psychological factors, and socioeconomic indicators.

### Clinical and economic implications

The preoperative baseline values identified in this study, including a mean EQ-5D-5L index of 0.43, may provide benchmark data for similar spine surgery populations in multi-ethnic Asian settings. Non-outpatient presentation was associated with lower baseline HRQoL and may help identify patients who could benefit from enhanced preoperative support and realistic expectation-setting. Observed ethnic disparities further emphasise the importance of culturally tailored education and counselling. The association between primary education or below and elevated anxiety or depression suggests that clinicians may wish to pay closer attention to psychological wellbeing in this subgroup.

From a health economic perspective, these preoperative EQ-5D values may inform future estimates of quality-adjusted life years gained from surgery in Singapore or similar Asian settings. Consistency of findings across the EQ-5D-3L and crosswalked EQ-5D-5L indices supports the use of mapped values in economic evaluation.

## Conclusion

Patients with degenerative lumbar spine conditions awaiting surgery experience substantial preoperative HRQoL impairment, particularly in the pain/discomfort and usual activities dimensions, with no significant variation across diagnostic groups. Non-outpatient presentation showed the largest association with lower baseline HRQoL and may help identify patients who would benefit from enhanced preoperative support. Ethnic and educational disparities highlight opportunities for culturally tailored counselling and psychological screening. Future longitudinal studies should evaluate whether these preoperative characteristics are associated with postoperative outcomes and whether targeted supportive interventions improve care.

## Supplementary Information

Below is the link to the electronic supplementary material.

Supplementary Material 1

## Data Availability

The data that support the findings of this study are available from the Spine Surgery Registry, National University Hospital, Singapore, but restrictions apply to their availability. Data may be made available from the corresponding author upon reasonable request and with permission of the registry custodian.
