# Correction: Factors associated with preoperative health-related quality of life in patients undergoing lumbar spine surgery: a multi-ethnic Asian cohort

**DOI:** 10.1007/s11136-026-04304-x

**Published:** 2026-07-13

**Authors:** Xun Li, Calvin Wei Jie Chern, Alex Quok An Teo, Jiong Hao Jonathan Tan, Annushiah Vasan Thakumar, Nan Luo, Hwee Weng Dennis Hey, Ling Jie Cheng

**Affiliations:** 1 School of Engineering, Computing, and Mathematics, Oxford Brookes University, Oxford, UK; 2 Alice Lee Centre for Nursing Studies, Yong Loo Lin School of Medicine, National University of Singapore, Singapore, Singapore; 3 Department of Orthopaedic Surgery, National University Hospital, Singapore, Singapore; 4 Department of Orthopaedic Surgery, Alexandra Hospital, Singapore, Singapore; 5 Yong Loo Lin School of Medicine, National University of Singapore, Singapore, Singapore; 6 School of Pharmacy, Faculty of Health & Medical Sciences, Taylor’s University, Subang Jaya, Selangor Malaysia; 7 Saw Swee Hock School of Public Health, National University of Singapore, Singapore, Singapore; 8 National Perinatal Epidemiology Unit, Nuffield Department of Women’s & Reproductive Health, University of Oxford, Oxford, UK

**Correction to: Quality of Life Research (2026) 35:144** https://doi.org/10.1007/s11136-026-04257-1

In this article, an error in the value-label assignments in the analytical Stata script resulted in incorrect labelling of categories for three variables in published Tables 1–4 and Supplementary Tables 2 and 3, and in corresponding sentences in the Abstract, Results, and Discussion. The error arose from a mismatch between Stata’s default alphabetical ordering produced by the encode command and the value labels subsequently defined in the script. The numeric counts and regression coefficients are correct; only the labels attached to the categories were incorrect.

The corrections are as follows.

## Sex

In Tables 1–4 and Supplementary Tables 2 and 3, all instances of “Female” and “Male” should be interchanged. The correct distribution is 615 (51.5%) male patients and 579 (48.5%) female patients. Consequently, the direction of the Sex effect is reversed in the regression analyses: male sex is associated with a higher EQ-5D-5L crosswalk index and lower odds of reporting pain/discomfort and anxiety/depression problems.

## Race/ethnicity

In Tables 1–4 and Supplementary Tables 2 and 3, all instances of “Malay” and “Indian” should be interchanged. The correct distribution is 145 (12.1%) Malay patients and 103 (8.6%) Indian patients. The overall conclusion that non-Chinese ethnicities have lower preoperative HRQoL than the Chinese majority remains unchanged.

## Education

In Tables 1–4 and Supplementary Tables 2 and 3, the three lower Education categories were cyclically rotated. The published “Primary or below” should read “Post-secondary/Diploma”; the published “Secondary” should read “Primary or below”; and the published “Post-secondary/Diploma” should read “Secondary”. The “University and above” category was correctly labelled. With corrected labels, the educational gradient is monotonic, with lower education associated with lower HRQoL, rather than the non-linear pattern described in the original publication.

The incorrect and corrected versions of Tables 1, 2, 3 and 4 are provided in this correction.

**Table 1:** Baseline characteristics and health-related quality of life of patients with degenerative lumbar spine conditions

Characteristic	*n* (%) or Mean (SD)
*Socio-demographic characteristics*	
Age (years), mean (SD)	58.1 (16.1)
Age category	
Young adults (<45 years)	245 (20.5)
Middle-aged (45–64 years)	443 (37.1)
Older adults (≥65 years)	506 (42.4)
Sex	
Male	615 (51.5)
Female	579 (48.5)
Race/ethnicity	
Chinese	839 (70.3)
Malay	145 (12.1)
Indian	103 (8.6)
Others	107 (9.0)
Education level	
Primary or below	237 (19.9)
Secondary	376 (31.5)
Post-secondary/Diploma	308 (25.8)
University and above	273 (22.9)
*Clinical characteristics*	
BMI (kg/m^2^), mean (SD)	26.3 (4.7)
BMI category (Asian cutoffs)	
Normal/Underweight (<23)	289 (24.2)
Overweight (23–27.4)	492 (41.2)
Obese (≥27.5)	413 (34.6)
Comorbidity status	
No comorbidities	377 (31.6)
≥1 comorbidity	817 (68.4)
Diagnosis	
Spinal stenosis	483 (40.5)
Prolapsed intervertebral disc	273 (22.9)
Spondylolisthesis	236 (19.8)
Degenerative disc disease	202 (16.9)
Spine level involvement	
L4/5	437 (36.6)
L4/5 and L5/S1	128 (10.7)
L5/S1	202 (16.9)
Mixed level	82 (6.9)
Others	344 (28.8)
*Healthcare and lifestyle factors*	
Presentation pathway	
Outpatient clinic	1118 (93.6)
Non-outpatient presentation	76 (6.4)
History of accident/trauma	
No	1131 (94.7)
Yes	63 (5.3)
Smoking history	
Never smoker	933 (78.1)
Former smoker	102 (8.5)
Current smoker	159 (13.3)
*Health-related quality of life*	
EQ-5D-5L crosswalk index, mean (SD)	0.43 (0.38)
EQ-5D-3L index, mean (SD)	0.48 (0.34)
EQ-5D-3L dimensions with any problems	
Mobility	885 (74.1)
Self-care	457 (38.3)
Usual activities	1007 (84.3)
Pain/discomfort	1118 (93.6)
Anxiety/depression	634 (53.1)

BMI, body mass index; EQ-5D-3L, 3-level EQ-5D; EQ-5D-5L, 5-level EQ-5D; SD, standard deviation

Data are presented as n (%) unless otherwise indicated. BMI categories based on WHO Asia-Pacific cutoffs. EQ-5D-5L crosswalk index derived using crosswalk algorithm from EQ-5D-3L responses. Presentation pathway at registry recruitment indicates the route by which patients entered the spine care pathway at recruitment: outpatient clinic vs. non-outpatient presentation (emergency department or direct inpatient recruitment). ‘Any problems’ includes levels 2–3 (some or extreme problems). Missing data: spine level (*n*=1)

**Table 2:** Univariate analyses of health-related quality of life by predictor categories

Characteristic	*n*	EQ-5D-5L crosswalk index	*p*
*Socio-demographic*			
Age category			0.851
Young adults (<45)	245	0.41 (0.42)	
Middle-aged (45–64)	443	0.43 (0.38)	
Older adults (≥65)	506	0.43 (0.36)	
Sex			0.003
Male	615	0.46 (0.38)	
Female	579	0.39 (0.38)	
Race/ethnicity			<0.001
Chinese	839	0.46 (0.36)	
Malay	145	0.32 (0.44)	
Indian	103	0.34 (0.41)	
Others	107	0.37 (0.42)	
Education level			<0.001
Primary or below	237	0.32 (0.39)	
Secondary	376	0.42 (0.37)	
Post-secondary/Diploma	308	0.46 (0.36)	
University and above	273	0.48 (0.39)	
*Clinical*			
BMI category			0.002
Normal/Underweight (<23)	289	0.43 (0.41)	
Overweight (23–27.4)	492	0.46 (0.35)	
Obese (≥27.5)	413	0.38 (0.39)	
Comorbidity status			0.360
No comorbidities	377	0.44 (0.40)	
≥1 comorbidity	817	0.42 (0.37)	
Diagnosis			0.526
Spinal stenosis	483	0.43 (0.37)	
Prolapsed intervertebral disc	273	0.40 (0.42)	
Spondylolisthesis	236	0.45 (0.35)	
DDD	202	0.41 (0.38)	
Spine level involvement			0.180
L4/5	437	0.44 (0.38)	
L4/5 and L5/S1	128	0.46 (0.35)	
L5/S1	202	0.40 (0.42)	
Mixed level	82	0.35 (0.38)	
Others	344	0.43 (0.37)	
*Healthcare and lifestyle*			
Presentation pathway			<0.001
Outpatient clinic	1118	0.45 (0.35)	
Non-outpatient presentation	76	0.04 (0.56)	
History of accident/trauma			<0.001
No	1131	0.43 (0.37)	
Yes	63	0.26 (0.46)	
Smoking history			0.071
Never smoker	933	0.44 (0.37)	
Former smoker	102	0.41 (0.38)	
Current smoker	159	0.36 (0.42)	

BMI, body mass index; DDD, degenerative disc disease

Data are presented as mean (SD). EQ-5D-5L crosswalk index derived using crosswalk algorithm. Presentation pathway at registry recruitment indicates the route by which patients entered the spine care pathway at recruitment: outpatient clinic vs. non-outpatient presentation (emergency department or direct inpatient recruitment)

*p*-values from independent samples t-test (binary variables) or one-way ANOVA (categorical variables with >2 groups)

**Table 3:** Hierarchical linear regression of factors associated with EQ-5D-5L crosswalk index

Characteristic	Model 1	Model 2	Model 3
	β (95% CI)	β (95% CI)	β (95% CI)
*Socio-demographic*			
Age category			
Young adults (< 45)	Ref	Ref	Ref
Middle-aged (45–64)	0.03 (−0.03, 0.09)	0.01 (−0.06, 0.08)	−0.01 (−0.07, 0.06)
Older adults (≥ 65)	0.03 (−0.03, 0.09)	0.00 (−0.08, 0.08)	−0.02 (−0.09, 0.06)
Sex			
Male	Ref	Ref	Ref
Female	-0.05 (-0.09, -0.01)*	-0.05 (-0.09, -0.00) *	-0.06 (-0.10, -0.01)*
Race/ethnicity			
Chinese	Ref	Ref	Ref
Malay	-0.14 (-0.21, -0.07) ***	-0.13 (-0.20, -0.06) ***	-0.08 (-0.15, -0.01) *
Indian	-0.14 (-0.22, -0.06) ***	-0.14 (-0.21, -0.06)***	-0.10 (-0.17, -0.02) *
Others	-0.14 (-0.22, -0.06) ***	−0.13 (− 0.21, − 0.06) **	−0.12 (− 0.20, − 0.05) **
Education level			
Primary or below	Ref	Ref	Ref
Secondary	0.11 (0.05, 0.17) ***	0.11 (0.05, 0.17) ***	0.10 (0.04, 0.16) ***
Post-secondary/Diploma	0.17 (0.10, 0.23)***	0.16 (0.10, 0.23)***	0.15 (0.09, 0.21) ***
University and above	0.19 (0.12, 0.26)***	0.19 (0.12, 0.26) ***	0.16 (0.09, 0.23) ***
*Clinical*			
BMI category			
Normal/Underweight (< 23)	—	Ref	Ref
Overweight (23–27.4)	—	0.03 (− 0.03, 0.08)	0.03 (− 0.03, 0.08)
Obese (≥ 27.5)	—	−0.03 (− 0.09, 0.02)	−0.03 (− 0.09, 0.02)
Comorbidity status			
No comorbidities	—	Ref	Ref
≥ 1 comorbidity	—	−0.03 (− 0.08, 0.03)	−0.03 (− 0.08, 0.02)
Diagnosis			
Spinal stenosis	—	Ref	Ref
Prolapsed intervertebral disc	—	−0.05 (− 0.12, 0.01)	−0.03 (− 0.10, 0.04)
Spondylolisthesis	—	0.01 (− 0.05, 0.07)	0.00 (− 0.06, 0.06)
DDD	—	−0.03 (− 0.09, 0.03)	−0.03 (− 0.09, 0.03)
Spine level involvement			
L4/5	—	Ref	Ref
L4/5 and L5/S1	—	0.02 (− 0.06, 0.09)	0.04 (− 0.03, 0.11)
L5/S1	—	−0.05 (− 0.11, 0.02)	−0.04 (− 0.10, 0.03)
Mixed level	—	−0.07 (− 0.16, 0.02)	−0.05 (− 0.13, 0.04)
Others	—	−0.01 (− 0.06, 0.05)	0.00 (− 0.05, 0.06)
*Healthcare and lifestyle*			
Presentation pathway			
Outpatient clinic	—	—	Ref
Non-outpatient presentation	—	—	−0.37 (− 0.46, − 0.28) ***
History of accident/trauma			
No	—	—	Ref
Yes	—	—	−0.11 (− 0.20, − 0.01) *
Smoking history			
Never smoker	—	—	Ref
Former smoker	—	—	−0.05 (− 0.13, 0.03)
Current smoker	—	—	−0.07 (− 0.13, − 0.00)
*Model fit indices*			
R^2^	0.057	0.069	0.132
Adjusted R^2^	0.049	0.054	0.115
AIC	1036.4	1040.4	964.8
BIC	1087.3	1142.1	1086.8
F statistic	7.88***	4.58***	7.74***
ΔR^2^	—	0.012	0.063

β, unstandardized regression coefficient; AIC, Akaike information criterion; BIC, Bayesian information criterion; BMI, body mass index; CI, confidence interval; DDD, degenerative disc disease

Model 1: Socio-demographic factors; Model 2: Model 1 + clinical factors; Model 3: Model 2 + healthcare and lifestyle factors. EQ-5D-5L crosswalk index derived using crosswalk algorithm. Presentation pathway at registry recruitment indicates the route by which patients entered the spine care pathway at recruitment: outpatient clinic vs. non-outpatient presentation (emergency department or direct inpatient recruitment)

ΔR^2^ represents change in R^2^ from previous model. *p < 0.05; **p < 0.01; ***p < 0.001

**Table 4:** Multivariable logistic regression of predictors of reporting any problems in EQ-5D-3L dimensions

Characteristic	Mobility	Self-care	Usual activities	Pain/discomfort	Anxiety/depression
	OR (95% CI)	OR (95% CI)	OR (95% CI)	OR (95% CI)	OR (95% CI)
*Socio-demographic*					
Age category					
Young adults (<45)	Ref	Ref	Ref	Ref	Ref
Middle-aged (45–64)	1.15 (0.76, 1.74)	1.02 (0.68, 1.51)	0.68 (0.40, 1.16)	0.48 (0.19, 1.16)	1.07 (0.74, 1.56)
Older adults (≥65)	1.41 (0.86, 2.32)	1.27 (0.80, 2.01)	0.57 (0.31, 1.07)	0.30 (0.11, 0.81) *	0.80 (0.51, 1.24)
Sex					
Male	Ref	Ref	Ref	Ref	Ref
Female	1.33 (0.99, 1.79)	1.29 (0.99, 1.69)	1.26 (0.89, 1.81)	1.90 (1.12, 3.22)*	1.40 (1.08, 1.81) **
Race/ethnicity					
Chinese	Ref	Ref	Ref	Ref	Ref
Malay	1.27 (0.79, 2.04)	1.20 (0.80, 1.78)	1.15 (0.64, 2.06)	3.14 (0.92, 10.66)	1.04 (0.70, 1.53)
Indian	1.76 (1.03, 3.02)	1.17 (0.75, 1.83)	1.37 (0.73, 2.59)	1.28 (0.51, 3.20)	1.08 (0.70, 1.67)
Others	1.28 (0.80, 2.07)	1.38 (0.89, 2.16)	3.40 (1.57, 7.37) **	2.23 (0.77, 6.52)	1.07 (0.70, 1.64)
Education level					
Primary or below	Ref	Ref	Ref	Ref	Ref
Secondary	0.53 (0.33, 0.85) **	0.61 (0.43, 0.86) **	0.60 (0.35, 1.03)	0.80 (0.38, 1.68)	0.65 (0.46, 0.92) *
Post-secondary/Diploma	0.37 (0.23, 0.60) ***	0.53 (0.37, 0.78) **	0.55 (0.31, 0.98) *	0.57 (0.26, 1.23)	0.51 (0.35, 0.74) ***
University and above	0.25 (0.15, 0.41) ***	0.41 (0.27, 0.62) ***	0.32 (0.18, 0.57) ***	0.52 (0.23, 1.18)	0.44 (0.29, 0.65) ***
*Clinical*					
BMI category					
Normal/Underweight (<23)	Ref	Ref	Ref	Ref	Ref
Overweight (23–27.4)	1.11 (0.78, 1.56)	1.13 (0.82, 1.56)	0.82 (0.54, 1.25)	1.44 (0.79, 2.60)	0.87 (0.64, 1.18)
Obese (≥27.5)	1.41 (0.97, 2.05)	1.49 (1.07, 2.08) *	1.15 (0.72, 1.82)	1.15 (0.61, 2.16)	0.89 (0.64, 1.22)
Comorbidity status					
No comorbidities	Ref	Ref	Ref	Ref	Ref
≥1 comorbidity	1.21 (0.87, 1.69)	1.09 (0.80, 1.48)	1.89 (1.28, 2.80) **	1.55 (0.87, 2.77)	1.24 (0.92, 1.66)
Diagnosis					
Spinal stenosis	Ref	Ref	Ref	Ref	Ref
Prolapsed intervertebral disc	1.11 (0.72, 1.69)	0.92 (0.62, 1.37)	0.80 (0.48, 1.34)	1.22 (0.55, 2.67)	1.31 (0.90, 1.91)
Spondylolisthesis	0.98 (0.67, 1.43)	0.80 (0.57, 1.12)	0.82 (0.53, 1.27)	1.28 (0.65, 2.51)	1.35 (0.98, 1.87)
DDD	1.97 (1.26, 3.08) **	0.95 (0.67, 1.36)	1.89 (1.08, 3.32) *	1.24 (0.62, 2.49)	1.16 (0.82, 1.65)
Spine level involvement					
L4/5	Ref	Ref	Ref	Ref	Ref
L4/5 and L5/S1	0.98 (0.61, 1.58)	0.74 (0.48, 1.15)	0.81 (0.46, 1.43)	1.87 (0.70, 5.02)	1.25 (0.83, 1.89)
L5/S1	0.89 (0.60, 1.33)	1.19 (0.82, 1.74)	1.27 (0.75, 2.15)	1.28 (0.56, 2.93)	1.25 (0.87, 1.80)
Mixed level	1.51 (0.78, 2.92)	1.13 (0.68, 1.87)	1.28 (0.59, 2.78)	1.06 (0.41, 2.76)	1.60 (0.96, 2.64)
Others	1.04 (0.73, 1.50)	0.85 (0.62, 1.17)	0.80 (0.52, 1.21)	1.03 (0.57, 1.87)	1.07 (0.79, 1.45)
*Healthcare and lifestyle*					
Presentation pathway					
Outpatient clinic	Ref	Ref	Ref	Ref	Ref
Non-outpatient presentation	1.50 (0.79, 2.82)	2.98 (1.79, 4.97) ***	2.03 (0.84, 4.92)	0.45 (0.19, 1.07)	1.57 (0.94, 2.60)
History of accident/trauma					
No	Ref	Ref	Ref	Ref	Ref
Yes	1.82 (0.88, 3.76)	1.20 (0.69, 2.08)	5.14 (1.22, 21.62) *	1.02 (0.30, 3.48)	0.96 (0.56, 1.65)
Smoking history					
Never smoker	Ref	Ref	Ref	Ref	Ref
Former smoker	1.39 (0.83, 2.34)	1.16 (0.74, 1.84)	1.77 (0.91, 3.47)	2.47 (0.85, 7.18)	1.28 (0.83, 1.99)
Current smoker	1.17 (0.75, 1.80)	1.29 (0.88, 1.88)	1.23 (0.70, 2.14)	1.26 (0.56, 2.87)	1.01 (0.70, 1.47)
Model fit indices					
Pseudo R^2^	0.071	0.046	0.074	0.061	0.032
AUC	0.68	0.64	0.70	0.70	0.62
AIC	1313.7	1563.3	1004.1	579.1	1645.1
LR χ^2^	97.04***	72.66***	76.62***	34.52	52.07***

AIC, Akaike information criterion; AUC, area under the receiver operating characteristic curve; BMI, body mass index; CI, confidence interval; DDD, degenerative disc disease; LR, likelihood ratio; OR, odds ratio

‘Any problems’ defined as reporting level 2 or 3 (some problems or extreme problems) vs. level 1 (no problems). Models adjusted for all variables shown. Presentation pathway at registry recruitment indicates the route by which patients entered the spine care pathway at recruitment: outpatient clinic vs. non-outpatient presentation (emergency department or direct inpatient recruitment)

*p<0.05; **p<0.01; ***p<0.001

**Table 1:** Baseline characteristics and health-related quality of life of patients with degenerative lumbar spine conditions

Characteristic	n (%) or Mean (SD)
*Socio-demographic characteristics*	
Age (years), mean (SD)	58.1 (16.1)
Age category	
Young adults (< 45 years)	245 (20.5)
Middle-aged (45–64 years)	443 (37.1)
Older adults (≥ 65 years)	506 (42.4)
Sex	
Male	615 (51.5)
Female	579 (48.5)
Race/ethnicity	
Chinese	839 (70.3)
Malay	145 (12.1)
Indian	103 (8.6)
Others	107 (9.0)
Education level	
Primary or below	237 (19.9)
Secondary	376 (31.5)
Post-secondary/Diploma	308 (25.8)
University and above	273 (22.9)
*Clinical characteristics*	
BMI (kg/m^2^), mean (SD)	26.3 (4.7)
BMI category (Asian cutoffs)	
Normal/Underweight (< 23)	289 (24.2)
Overweight (23–27.4)	492 (41.2)
Obese (≥ 27.5)	413 (34.6)
Comorbidity status	
No comorbidities	377 (31.6)
≥ 1 comorbidity	817 (68.4)
Diagnosis	
Spinal stenosis	483 (40.5)
Prolapsed intervertebral disc	273 (22.9)
Spondylolisthesis	236 (19.8)
Degenerative disc disease	202 (16.9)
Spine level involvement	
L4/5	437 (36.6)
L4/5 and L5/S1	128 (10.7)
L5/S1	202 (16.9)
Mixed level	82 (6.9)
Others	344 (28.8)
Healthcare and lifestyle factors	
Presentation pathway	
Outpatient clinic	1,118 (93.6)
Non-outpatient presentation	76 (6.4)
History of accident/trauma	
No	1,131 (94.7)
Yes	63 (5.3)
Smoking history	
Never smoker	933 (78.1)
Former smoker	102 (8.5)
Current smoker	159 (13.3)
*Health-related quality of life*	
EQ-5D-5L crosswalk index, mean (SD)	0.43 (0.38)
EQ-5D-3L index, mean (SD)	0.48 (0.34)
EQ-5D-3L dimensions with any problems	
Mobility	885 (74.1)
Self-care	457 (38.3)
Usual activities	1,007 (84.3)
Pain/discomfort	1,118 (93.6)
Anxiety/depression	634 (53.1)

BMI, body mass index; EQ-5D-5L, EuroQol 5-Dimension 5-Level; EQ-5D-3L, EuroQol 5-Dimension 3-Level; SD, standard deviation

Data are presented as n (%) unless otherwise indicated. BMI categories are based on WHO Asia–Pacific cutoffs. The EQ-5D-5L crosswalk index is derived using the van Hout 2021 crosswalk algorithm from EQ-5D-3L responses. Presentation pathway at registry recruitment indicates the route by which patients entered the spine care pathway: outpatient clinic vs non-outpatient presentation (emergency department or direct inpatient recruitment). “Any problems” includes levels 2 and 3 (some or extreme problems). Missing data: spine level (n = 1)

**Table 2:** Univariate analyses of health-related quality of life by predictor categories

Characteristic	n	EQ-5D-5L crosswalk index	*p*
*Socio-demographic*			
Age category			0.851
Young adults (< 45)	245	0.41 (0.42)	
Middle-aged (45–64)	443	0.43 (0.38)	
Older adults (≥ 65)	506	0.43 (0.36)	
Sex			0.003
Male	615	0.46 (0.38)	
Female	579	0.39 (0.38)	
Race/ethnicity			<0.001
Chinese	839	0.46 (0.36)	
Malay	145	0.32 (0.44)	
Indian	103	0.34 (0.41)	
Others	107	0.37 (0.42)	
Education level			<0.001
Primary or below	237	0.32 (0.39)	
Secondary	376	0.42 (0.37)	
Post-secondary/Diploma	308	0.46 (0.36)	
University and above	273	0.48 (0.39)	
*Clinical*			
BMI category			0.002
Normal/Underweight (< 23)	289	0.43 (0.41)	
Overweight (23–27.4)	492	0.46 (0.35)	
Obese (≥ 27.5)	413	0.38 (0.39)	
Comorbidity status			0.360
No comorbidities	377	0.44 (0.40)	
≥ 1 comorbidity	817	0.42 (0.37)	
Diagnosis			0.526
Spinal stenosis	483	0.43 (0.37)	
Prolapsed intervertebral disc	273	0.40 (0.42)	
Spondylolisthesis	236	0.45 (0.35)	
DDD	202	0.41 (0.38)	
Spine level involvement			0.180
L4/5	437	0.44 (0.38)	
L4/5 and L5/S1	128	0.46 (0.35)	
L5/S1	202	0.40 (0.42)	
Mixed level	82	0.35 (0.38)	
Others	344	0.43 (0.37)	
*Healthcare and lifestyle*			
Presentation pathway			<0.001
Outpatient clinic	1,118	0.45 (0.35)	
Non-outpatient presentation	76	0.04 (0.56)	
History of accident/trauma			<0.001
No	1,131	0.43 (0.37)	
Yes	63	0.26 (0.46)	
Smoking history			0.071
Never smoker	933	0.44 (0.37)	
Former smoker	102	0.41 (0.38)	
Current smoker	159	0.36 (0.42)	

BMI, body mass index; DDD, degenerative disc disease; EQ-5D-5L, EuroQol 5-Dimension 5-Level

Data are presented as mean (SD). The EQ-5D-5L crosswalk index is derived using the van Hout 2021 crosswalk algorithm. p-values are from the independent samples t-test (binary variables) or one-way ANOVA (categorical variables with more than two groups)

**Table 3:** Hierarchical linear regression of factors associated with EQ-5D-5L crosswalk index

Characteristic	Model 1 β (95% CI)	Model 2 β (95% CI)	Model 3 β (95% CI)
*Socio-demographic*			
Age category			
Young adults (< 45)	Ref	Ref	Ref
Middle-aged (45–64)	0.03 (− 0.03, 0.09)	0.01 (− 0.06, 0.08)	− 0.00 (− 0.07, 0.06)
Older adults (≥ 65)	0.03 (− 0.03, 0.09)	0.00 (− 0.08, 0.08)	− 0.02 (− 0.09, 0.06)
Sex			
Male	Ref	Ref	Ref
Female	− 0.05 (− 0.09, − 0.01)*	− 0.05 (− 0.09, − 0.00)*	− 0.06 (− 0.10, − 0.01)*
Race/ethnicity			
Chinese	Ref	Ref	Ref
Malay	− 0.14 (− 0.21, − 0.07)***	− 0.13 (− 0.20, − 0.06)***	− 0.08 (− 0.15, − 0.01)*
Indian	− 0.14 (− 0.22, − 0.06)***	− 0.14 (− 0.21, − 0.06)***	− 0.10 (− 0.17, − 0.02)*
Others	− 0.14 (− 0.22, − 0.06)***	− 0.13 (− 0.21, − 0.06)***	− 0.12 (− 0.20, − 0.05)**
Education level			
Primary or below	Ref	Ref	Ref
Secondary	0.11 (0.05, 0.17)***	0.11 (0.05, 0.17)***	0.10 (0.04, 0.16)**
Post-secondary/Diploma	0.17 (0.10, 0.23)***	0.16 (0.10, 0.23)***	0.15 (0.09, 0.21)***
University and above	0.19 (0.12, 0.26)***	0.19 (0.12, 0.26)***	0.16 (0.09, 0.23)***
*Clinical*			
BMI category			
Normal/Underweight (< 23)	–	Ref	Ref
Overweight (23–27.4)	–	0.03 (− 0.03, 0.08)	0.03 (− 0.03, 0.08)
Obese (≥ 27.5)	–	− 0.03 (− 0.09, 0.02)	− 0.03 (− 0.09, 0.02)
Comorbidity status			
No comorbidities	–	Ref	Ref
≥ 1 comorbidity	–	− 0.03 (− 0.08, 0.03)	− 0.03 (− 0.08, 0.02)
Diagnosis			
Spinal stenosis	–	Ref	Ref
Prolapsed intervertebral disc	–	− 0.05 (− 0.12, 0.01)	− 0.03 (− 0.10, 0.04)
Spondylolisthesis	–	0.01 (− 0.05, 0.07)	0.00 (− 0.06, 0.06)
DDD	–	− 0.03 (− 0.09, 0.03)	− 0.03 (− 0.09, 0.03)
Spine level involvement			
L4/5	–	Ref	Ref
L4/5 and L5/S1	–	0.02 (− 0.06, 0.09)	0.04 (− 0.03, 0.11)
L5/S1	–	− 0.05 (− 0.11, 0.02)	− 0.04 (− 0.10, 0.03)
Mixed level	–	− 0.07 (− 0.16, 0.02)	− 0.05 (− 0.13, 0.04)
Others	–	− 0.01 (− 0.06, 0.05)	0.00 (− 0.05, 0.06)
*Healthcare and lifestyle*			
Presentation pathway			
Outpatient clinic	–	–	Ref
Non-outpatient presentation	–	–	− 0.37 (− 0.46, − 0.28)***
History of accident/trauma			
No	–	–	Ref
Yes	–	–	− 0.11 (− 0.20, − 0.01)*
Smoking history			
Never smoker	–	–	Ref
Former smoker	–	–	− 0.05 (− 0.13, 0.03)
Current smoker	–	–	− 0.07 (− 0.13, − 0.00)*
*Model fit indices*			
R^2^	0.057	0.069	0.132
Adjusted R^2^	0.05	0.054	0.115
AIC	1036	1040.4	964.8
BIC	1086.9	1142.1	1086.8

β, unstandardised regression coefficient; AIC, Akaike information criterion; BIC, Bayesian information criterion; BMI, body mass index; CI, confidence interval; DDD, degenerative disc disease

Model 1 includes socio-demographic factors; Model 2 adds clinical factors; Model 3 adds healthcare and lifestyle factors. Outcome: EQ-5D-5L crosswalk index (van Hout 2021 algorithm)

Significance levels: *p < 0.05; **p < 0.01; ***p < 0.001

**Table 4:** Multivariable logistic regression of predictors of reporting any problems in EQ-5D-3L dimensions

Characteristic	Mobility	Self-care	Usual activities	Pain/discomfort	Anxiety/depression
	OR (95% CI)	OR (95% CI)	OR (95% CI)	OR (95% CI)	OR (95% CI)
*Socio-demographic*					
Age category					
Young adults (< 45)	Ref	Ref	Ref	Ref	Ref
Middle-aged (45–64)	1.15 (0.76, 1.74)	1.02 (0.68, 1.51)	0.68 (0.40, 1.16)	0.48 (0.19, 1.16)	1.07 (0.74, 1.56)
Older adults (≥ 65)	1.41 (0.86, 2.32)	1.27 (0.80, 2.01)	0.57 (0.31, 1.07)	0.30 (0.11, 0.81)*	0.80 (0.51, 1.24)
Sex					
Male	Ref	Ref	Ref	Ref	Ref
Female	1.33 (0.99, 1.79)	1.29 (0.99, 1.69)	1.26 (0.89, 1.81)	1.90 (1.12, 3.22)*	1.40 (1.08, 1.81)**
Race/ethnicity					
Chinese	Ref	Ref	Ref	Ref	Ref
Malay	1.27 (0.79, 2.04)	1.20 (0.80, 1.78)	1.15 (0.64, 2.06)	3.14 (0.92, 10.66)	1.04 (0.70, 1.53)
Indian	1.76 (1.03, 3.02)*	1.17 (0.75, 1.83)	1.37 (0.73, 2.59)	1.28 (0.51, 3.20)	1.08 (0.70, 1.67)
Others	1.28 (0.80, 2.07)	1.38 (0.89, 2.16)	3.40 (1.57, 7.37)**	2.23 (0.77, 6.52)	1.07 (0.70, 1.64)
Education level					
Primary or below	Ref	Ref	Ref	Ref	Ref
Secondary	0.53 (0.33, 0.85)**	0.61 (0.43, 0.86)**	0.60 (0.35, 1.03)	0.80 (0.38, 1.68)	0.65 (0.46, 0.92)*
Post-secondary/Diploma	0.37 (0.23, 0.60)***	0.53 (0.37, 0.78)**	0.55 (0.31, 0.98)*	0.57 (0.26, 1.23)	0.51 (0.35, 0.74)***
University and above	0.25 (0.15, 0.41)***	0.41 (0.27, 0.62)***	0.32 (0.18, 0.57)***	0.52 (0.23, 1.18)	0.44 (0.29, 0.65)***
*Clinical*					
BMI category					
Normal/Underweight (< 23)	Ref	Ref	Ref	Ref	Ref
Overweight (23–27.4)	1.11 (0.78, 1.56)	1.13 (0.82, 1.56)	0.82 (0.54, 1.25)	1.44 (0.79, 2.60)	0.87 (0.64, 1.18)
Obese (≥ 27.5)	1.41 (0.97, 2.05)	1.49 (1.07, 2.08)*	1.15 (0.72, 1.82)	1.15 (0.61, 2.16)	0.89 (0.64, 1.22)
Comorbidity status					
No comorbidities	Ref	Ref	Ref	Ref	Ref
≥ 1 comorbidity	1.21 (0.87, 1.69)	1.09 (0.80, 1.48)	1.89 (1.28, 2.80)**	1.55 (0.87, 2.77)	1.24 (0.92, 1.66)
Diagnosis					
Spinal stenosis	Ref	Ref	Ref	Ref	Ref
Prolapsed intervertebral disc	1.11 (0.72, 1.69)	0.92 (0.62, 1.37)	0.80 (0.48, 1.34)	1.22 (0.55, 2.67)	1.31 (0.90, 1.91)
Spondylolisthesis	0.98 (0.67, 1.43)	0.80 (0.57, 1.12)	0.82 (0.53, 1.27)	1.28 (0.65, 2.51)	1.35 (0.98, 1.87)
DDD	1.97 (1.26, 3.08)**	0.95 (0.67, 1.36)	1.89 (1.08, 3.32)*	1.24 (0.62, 2.49)	1.16 (0.82, 1.65)
Spine level involvement					
L4/5	Ref	Ref	Ref	Ref	Ref
L4/5 and L5/S1	0.98 (0.61, 1.58)	0.74 (0.48, 1.15)	0.81 (0.46, 1.43)	1.87 (0.70, 5.02)	1.25 (0.83, 1.89)
L5/S1	0.89 (0.60, 1.33)	1.19 (0.82, 1.74)	1.27 (0.75, 2.15)	1.28 (0.56, 2.93)	1.25 (0.87, 1.80)
Mixed level	1.51 (0.78, 2.92)	1.13 (0.68, 1.87)	1.28 (0.59, 2.78)	1.06 (0.41, 2.76)	1.60 (0.96, 2.64)
Others	1.04 (0.73, 1.50)	0.85 (0.62, 1.17)	0.80 (0.52, 1.21)	1.03 (0.57, 1.87)	1.07 (0.79, 1.45)
*Healthcare and lifestyle*					
Presentation pathway					
Outpatient clinic	Ref	Ref	Ref	Ref	Ref
Non-outpatient presentation	1.50 (0.79, 2.82)	2.98 (1.79, 4.97)***	2.03 (0.84, 4.92)	0.45 (0.19, 1.07)	1.57 (0.94, 2.60)
History of accident/trauma					
No	Ref	Ref	Ref	Ref	Ref
Yes	1.82 (0.88, 3.76)	1.20 (0.69, 2.08)	5.14 (1.22, 21.62)*	1.02 (0.30, 3.48)	0.96 (0.56, 1.65)
Smoking history					
Never smoker	Ref	Ref	Ref	Ref	Ref
Former smoker	1.39 (0.83, 2.34)	1.16 (0.74, 1.84)	1.77 (0.91, 3.47)	2.47 (0.85, 7.18)	1.28 (0.83, 1.99)
Current smoker	1.17 (0.75, 1.80)	1.29 (0.88, 1.88)	1.23 (0.70, 2.14)	1.26 (0.56, 2.87)	1.01 (0.70, 1.47)
*Model fit indices*					
Pseudo R^2^	0.071	0.046	0.074	0.061	0.032
AUC	0.68	0.64	0.7	0.7	0.62
AIC	1313.7	1563.3	1004.1	579.1	1645.1

AIC, Akaike information criterion; AUC, area under the receiver operating characteristic curve; BMI, body mass index; CI, confidence interval; DDD, degenerative disc disease; OR, odds ratio

“Any problems” is defined as reporting level 2 or 3 (some or extreme problems) versus level 1 (no problems) on the EQ-5D-3L. Models are adjusted for all variables shown

Significance levels: *p < 0.05; **p < 0.01; ***p < 0.001

The Abstract Results subsection, Results section, and two paragraphs of the Discussion, namely the Female sex and Education paragraphs, are revised below to reflect the corrected analyses. The Methods, Introduction, Plain English Summary, Conclusions, and reference list are unchanged.

## Revised Abstract — Results subsection

The Abstract Results subsection should read: “Mean age was 58.1 years (SD 16.1); 51.5% were male. Mean EQ-5D-5L crosswalk index was 0.43 (SD 0.38). Pain/discomfort (93.6%) and usual activities problems (84.3%) were most commonly reported. Lower EQ-5D scores were independently associated with non-outpatient presentation (β = − 0.37), non-Chinese ethnicity, primary education or below relative to higher levels, and history of accident or trauma (β = − 0.11). Dimension-level analyses showed primary education or below was associated with higher odds of problems across mobility, self-care, usual activities, and anxiety/depression compared with higher education levels, with a clear monotonic gradient. Female sex was associated with higher odds of pain/discomfort and anxiety/depression problems compared with male sex. Non-outpatient presentation was associated with markedly higher odds of self-care problems (OR = 2.99).”

## Revised Results section

The following sentences in the Results section are revised:

“Of 1,194 patients included in the analytic sample, 615 (51.5%) were male and 579 (48.5%) were female.” This replaces the original sentence stating the converse.

“In Model 1 (sociodemographic factors only), male sex was associated with higher EQ-5D-5L index (β = 0.05, 95% CI: 0.01 to 0.09, *p* < 0.05). Compared with primary education or below, higher educational attainment was associated with progressively higher EQ-5D-5L index, in a monotonic gradient: secondary (β = 0.11, 95% CI: 0.05 to 0.17, *p* < 0.001), post-secondary or Diploma (β = 0.17, 95% CI: 0.10 to 0.23, *p* < 0.001), and university and above (β = 0.19, 95% CI: 0.12 to 0.26, *p *< 0.001).” This replaces the original sentence describing female sex as protective and secondary education as deleterious.

“For mobility, higher education was associated with progressively lower odds of reporting problems compared with primary education or below, in a clear monotonic gradient: secondary (OR = 0.53, 95% CI: 0.33 to 0.85, *p* < 0.01), post-secondary or Diploma (OR = 0.37, 95% CI: 0.23 to 0.60, *p* < 0.001), and university or above (OR = 0.25, 95% CI: 0.15 to 0.41, *p* < 0.001).” This replaces the original sentence describing secondary education as associated with higher odds of mobility problems.

“For pain/discomfort, females had higher odds of reporting problems compared with males (OR = 1.90, 95% CI: 1.12 to 3.22, *p* < 0.05).” This replaces the original sentence describing female sex as associated with lower odds of pain/discomfort.

“For anxiety/depression, female sex was associated with increased odds of reporting problems (OR = 1.40, 95% CI: 1.08 to 1.81, *p* < 0.05). Higher education was associated with progressively reduced odds of anxiety/depression problems compared with primary education or below: secondary (OR = 0.65, 95% CI: 0.46 to 0.92, *p* < 0.05), post-secondary or Diploma (OR=0.51, 95% CI: 0.35 to 0.74, p<0.001), and university or above (OR = 0.44, 95% CI: 0.29 to 0.65, *p* < 0.001).”This replaces the original sentence describing female sex as protective and secondary education as deleterious.

## Revised Discussion — Female sex paragraph

The Female sex paragraph in the Discussion should be replaced with the following:

“Female sex was associated with higher odds of reporting pain/discomfort and anxiety/depression problems and with marginally lower preoperative EQ-5D index scores. These findings are consistent with prior literature documenting that women undergoing lumbar spine surgery often present with greater preoperative pain and psychological distress [38, 39, 40, 41]. Possible explanations include sex-based differences in pain perception and reporting [21], cumulative musculoskeletal burden related to occupational and caregiving roles, and culturally shaped help-seeking patterns where women may delay surgical consultation until symptoms become more severe, with evidence that help-seeking tendencies differ between Western and Asian populations [42]. These findings suggest that female patients awaiting lumbar spine surgery may benefit from targeted preoperative pain management and psychological support, and that referral pathways should be examined to ensure timely access for women presenting with degenerative lumbar conditions.”

## Revised Discussion — Education paragraph

The Education paragraph in the Discussion should be replaced with the following:

“A clear monotonic gradient was observed between higher education and better preoperative HRQoL. Compared with primary education or below, all higher education categories were associated with lower odds of problems across mobility, self-care, usual activities, and anxiety/depression, with the strongest effects in the university-educated group. This pattern is consistent with a substantial body of evidence linking higher educational attainment to better health literacy, more effective health-seeking behaviour, and lower disease burden in chronic conditions [19, 20]. Patients with primary education or below also had higher odds of anxiety or depression problems, suggesting a potential role for psychological screening and culturally adapted patient education in this subgroup. Clinicians should consider the role of educational background in shaping patient expectations and engagement with preoperative care.”

## Findings unchanged

The principal study conclusions remain unchanged. Non-outpatient presentation has the largest association with poorer preoperative HRQoL (β = − 0.37, *p* < 0.001). History of accident or trauma is associated with poorer HRQoL. Non-Chinese ethnicities have lower preoperative HRQoL than the Chinese majority. The Plain English Summary and Conclusions remain accurate.

The original article has been corrected.

## Supplementary Information

Below is the link to the electronic supplementary material.

Supplementary Material 1

