# Supplementary material for: Factors associated with preoperative health-related quality of life in patients undergoing lumbar spine surgery: a multi-ethnic Asian cohort
Source: Qual Life Res. 2026 May 3;35(6):144. doi: 10.1007/s11136-026-04257-1 (PMC13136196; doi:10.1007/s11136-026-04257-1)
Supplement: Supplementary file 1 — Supplementary Material 1 [file 11136_2026_4257_MOESM1_ESM.docx]

**Supplementary Table 1** Model fit statistics for hierarchical regression models

**Panel A: Linear regression models**

| **Model** | **N** | **R²** | **Adj. R²** | **RMSE** | **AIC** | **BIC** |
| --- | --- | --- | --- | --- | --- | --- |
| ***EQ-5D-5L crosswalk index*** |  |  |  |  |  |  |
| Model 1: Socio-demographic | 1,194 | 0.057 | 0.049 | 0.372 | 1036.4 | 1087.3 |
| Model 2: + Clinical | 1,193 | 0.069 | 0.054 | 0.371 | 1040.4 | 1142.1 |
| Model 3: + Healthcare/lifestyle | 1,193 | 0.132 | 0.115 | 0.359 | 964.8 | 1086.8 |

**Panel B: Logistic regression models**

| **Model** | **N** | **Pseudo R²** | **AIC** | **BIC** | **AUC** |
| --- | --- | --- | --- | --- | --- |
| ***Mobility*** |  |  |  |  |  |
| Model 1: Socio-demographic | 1,194 | 0.050 | 1316.8 | 1367.7 | 0.65 |
| Model 2: + Clinical | 1,193 | 0.066 | 1312.6 | 1414.3 | 0.68 |
| Model 3: + Healthcare/lifestyle | 1,193 | 0.071 | 1313.7 | 1435.7 | 0.68 |
| ***Self-care*** |  |  |  |  |  |
| Model 1: Socio-demographic | 1,194 | 0.024 | 1571.0 | 1621.8 | 0.60 |
| Model 2: + Clinical | 1,193 | 0.033 | 1576.2 | 1677.9 | 0.62 |
| Model 3: + Healthcare/lifestyle | 1,193 | 0.046 | 1563.3 | 1685.4 | 0.64 |
| ***Usual activities*** |  |  |  |  |  |
| Model 1: Socio-demographic | 1,194 | 0.033 | 1022.4 | 1073.3 | 0.63 |
| Model 2: + Clinical | 1,193 | 0.060 | 1011.2 | 1112.9 | 0.68 |
| Model 3: + Healthcare/lifestyle | 1,193 | 0.074 | 1004.1 | 1126.1 | 0.70 |
| ***Pain/discomfort*** |  |  |  |  |  |
| Model 1: Socio-demographic | 1,194 | 0.038 | 564.3 | 615.2 | 0.66 |
| Model 2: + Clinical | 1,193 | 0.050 | 577.3 | 679.0 | 0.68 |
| Model 3: + Healthcare/lifestyle | 1,193 | 0.061 | 579.1 | 701.1 | 0.70 |
| ***Anxiety/depression*** |  |  |  |  |  |
| Model 1: Socio-demographic | 1,194 | 0.022 | 1635.0 | 1685.9 | 0.60 |
| Model 2: + Clinical | 1,193 | 0.029 | 1641.5 | 1743.2 | 0.62 |
| Model 3: + Healthcare/lifestyle | 1,193 | 0.032 | 1645.1 | 1767.1 | 0.62 |

*Abbreviations:* Adj., adjusted; AIC, Akaike information criterion; AUC, area under the receiver operating characteristic curve; BIC, Bayesian information criterion; RMSE, root mean square error. *Notes:* Model 1 includes socio-demographic factors (age, sex, race/ethnicity, education). Model 2 adds clinical factors (BMI, comorbidity status, diagnosis, spine level involvement). Model 3 adds healthcare and lifestyle factors (presentation pathway at registry recruitment, accident history, smoking history). For linear regression, lower AIC/BIC and higher R² indicate better model fit. For logistic regression, lower AIC/BIC and higher Pseudo R²/AUC indicate better model fit.

**Supplementary Table 2** Sensitivity analysis: Hierarchical linear regression of predictors of EQ-5D-3L index

| **Characteristic** | **Model 1** | **Model 2** | **Model 3** |
| --- | --- | --- | --- |
|  | ***β (95% CI)*** | ***β (95% CI)*** | ***β (95% CI)*** |
| **Socio-demographic** |  |  |  |
| Age category |  |  |  |
| Young adults (<45) | Ref | Ref | Ref |
| Middle-aged (45–64) | 0.03 (−0.02, 0.09) | 0.01 (−0.05, 0.07) | −0.01 (−0.07, 0.05) |
| Older adults (≥65) | 0.04 (−0.02, 0.09) | 0.00 (−0.07, 0.07) | −0.01 (−0.08, 0.06) |
| Sex |  |  |  |
| Male | Ref | Ref | Ref |
| Female | 0.04 (0.00, 0.08) * | 0.04 (0.00, 0.08) * | 0.05 (0.01, 0.09) * |
| Race/ethnicity |  |  |  |
| Chinese | Ref | Ref | Ref |
| Malay | −0.13 (−0.20, −0.06) *** | −0.13 (−0.20, −0.06) *** | −0.09 (−0.16, −0.03) ** |
| Indian | −0.14 (−0.20, −0.08) *** | −0.13 (−0.19, −0.06) *** | −0.09 (−0.15, −0.03) ** |
| Others | −0.12 (−0.19, −0.05) ** | −0.12 (−0.18, −0.05) ** | −0.11 (−0.17, −0.04) ** |
| Education level |  |  |  |
| Primary or below | Ref | Ref | Ref |
| Secondary | −0.13 (−0.19, −0.07) *** | −0.13 (−0.19, −0.07) *** | −0.11 (−0.17, −0.06) *** |
| Post-secondary/Diploma | −0.05 (−0.10, 0.00) | −0.05 (−0.10, 0.00) | −0.04 (−0.09, 0.01) |
| University and above | −0.01 (−0.06, 0.04) | −0.01 (−0.06, 0.05) | −0.01 (−0.07, 0.04) |
| **Clinical** |  |  |  |
| BMI category |  |  |  |
| Normal/Underweight (<23) | — | Ref | Ref |
| Overweight (23–27.4) | — | 0.03 (−0.02, 0.08) | 0.03 (−0.02, 0.07) |
| Obese (≥27.5) | — | −0.02 (−0.07, 0.03) | −0.02 (−0.07, 0.03) |
| Comorbidity status |  |  |  |
| No comorbidities | — | Ref | Ref |
| ≥1 comorbidity | — | −0.01 (−0.06, 0.04) | −0.02 (−0.06, 0.03) |
| Diagnosis |  |  |  |
| Spinal stenosis | — | Ref | Ref |
| Prolapsed intervertebral disc | — | −0.06 (−0.12, 0.00) | −0.04 (−0.10, 0.02) |
| Spondylolisthesis | — | 0.00 (−0.05, 0.05) | −0.01 (−0.06, 0.04) |
| DDD | — | −0.02 (−0.07, 0.04) | −0.02 (−0.07, 0.04) |
| Spine level involvement |  |  |  |
| L4/5 | — | Ref | Ref |
| L4/5 and L5/S1 | — | 0.00 (−0.06, 0.07) | 0.02 (−0.04, 0.09) |
| L5/S1 | — | −0.04 (−0.10, 0.02) | −0.03 (−0.09, 0.02) |
| Mixed level | — | −0.06 (−0.14, 0.02) | −0.04 (−0.12, 0.03) |
| Others | — | −0.02 (−0.07, 0.03) | −0.01 (−0.05, 0.04) |
| **Healthcare and lifestyle** |  |  |  |
| Presentation pathway |  |  |  |
| Outpatient clinic | — | — | Ref |
| Non-outpatient presentation | — | — | −0.33 (−0.41, −0.26) *** |
| History of accident/trauma |  |  |  |
| No | — | — | Ref |
| Yes | — | — | −0.09 (−0.17, −0.01) * |
| Smoking history |  |  |  |
| Never smoker | — | — | Ref |
| Former smoker | — | — | 0.04 (−0.03, 0.11) |
| Current smoker | — | — | −0.02 (−0.10, 0.06) |
| **Model fit indices** |  |  |  |
| R² | 0.052 | 0.063 | 0.128 |
| Adjusted R² | 0.045 | 0.048 | 0.111 |
| RMSE | 0.327 | 0.327 | 0.316 |
| AIC | 732.0 | 737.9 | 673.0 |
| BIC | 782.9 | 839.6 | 795.0 |
| ΔR² | — | 0.011 | 0.065 |

*Abbreviations:* β, unstandardized regression coefficient; AIC, Akaike information criterion; BIC, Bayesian information criterion; BMI, body mass index; CI, confidence interval; DDD, degenerative disc disease; RMSE, root mean square error. *Notes:* Model 1: Socio-demographic factors; Model 2: Model 1 + clinical factors; Model 3: Model 2 + healthcare and lifestyle factors. EQ-5D-3L index calculated using Singapore value set. Presentation pathway at registry recruitment indicates the route by which patients entered the spine care pathway at recruitment: outpatient clinic vs non-outpatient presentation (emergency department or direct inpatient recruitment). ΔR² represents change in R² from previous model. *p < 0.05; **p < 0.01; ***p < 0.001

**Supplementary Table 3** Comparison of full model results: EQ-5D-5L ^cw^ crosswalk vs EQ-5D-3L index

| **Characteristic** | **EQ-5D-5L crosswalk index** | **EQ-5D-3L Index** |
| --- | --- | --- |
|  | ***β (95% CI)*** | ***β (95% CI)*** |
| **Socio-demographic** |  |  |
| Age category |  |  |
| Young adults (<45) | Ref | Ref |
| Middle-aged (45–64) | −0.01 (−0.07, 0.06) | −0.01 (−0.07, 0.05) |
| Older adults (≥65) | −0.02 (−0.09, 0.06) | −0.01 (−0.08, 0.06) |
| Sex |  |  |
| Male | Ref | Ref |
| Female | 0.06 (0.01, 0.10) * | 0.05 (0.01, 0.09) * |
| Race/ethnicity |  |  |
| Chinese | Ref | Ref |
| Malay | −0.10 (−0.17, −0.02) * | −0.09 (−0.16, −0.03) ** |
| Indian | −0.08 (−0.15, −0.01) * | −0.09 (−0.15, −0.03) ** |
| Others | −0.12 (−0.20, −0.05) ** | −0.11 (−0.17, −0.04) ** |
| Education level |  |  |
| Primary or below | Ref | Ref |
| Secondary | −0.15 (−0.21, −0.09) *** | −0.11 (−0.17, −0.06) *** |
| Post-secondary/Diploma | −0.05 (−0.11, 0.01) | −0.04 (−0.09, 0.01) |
| University and above | 0.01 (−0.05, 0.08) | −0.01 (−0.07, 0.04) |
| **Clinical** |  |  |
| BMI category |  |  |
| Normal/Underweight (<23) | Ref | Ref |
| Overweight (23–27.4) | 0.03 (−0.03, 0.08) | 0.03 (−0.02, 0.07) |
| Obese (≥27.5) | −0.03 (−0.09, 0.02) | −0.02 (−0.07, 0.03) |
| Comorbidity status |  |  |
| No comorbidities | Ref | Ref |
| ≥1 comorbidity | −0.03 (−0.08, 0.02) | −0.02 (−0.06, 0.03) |
| Diagnosis |  |  |
| Spinal stenosis | Ref | Ref |
| Prolapsed disc | −0.03 (−0.10, 0.04) | −0.04 (−0.10, 0.02) |
| Spondylolisthesis | 0.00 (−0.06, 0.06) | −0.01 (−0.06, 0.04) |
| DDD | −0.03 (−0.09, 0.03) | −0.02 (−0.07, 0.04) |
| Spine level involvement |  |  |
| L4/5 | Ref | Ref |
| L4/5 and L5/S1 | 0.04 (−0.03, 0.11) | 0.02 (−0.04, 0.09) |
| L5/S1 | −0.04 (−0.10, 0.03) | −0.03 (−0.09, 0.02) |
| Mixed level | −0.05 (−0.13, 0.04) | −0.04 (−0.12, 0.03) |
| Others | 0.00 (−0.05, 0.06) | −0.01 (−0.05, 0.04) |
| **Healthcare and lifestyle** |  |  |
| Presentation pathway |  |  |
| Outpatient clinic | Ref | Ref |
| Non-outpatient presentation | −0.37 (−0.46, −0.28) *** | −0.33 (−0.41, −0.26) *** |
| History of accident/trauma |  |  |
| No | Ref | Ref |
| Yes | −0.11 (−0.20, −0.01) * | −0.09 (−0.17, −0.01) * |
| Smoking history |  |  |
| Never smoker | Ref | Ref |
| Former smoker | −0.05 (−0.13, 0.03) | −0.04 (−0.11, 0.03) |
| Current smoker | −0.07 (−0.13, −0.00) | −0.06 (−0.12, −0.00) |
| **Model fit indices** |  |  |
| R² | 0.132 | 0.128 |
| Adjusted R² | 0.115 | 0.111 |
| RMSE | 0.359 | 0.316 |
| AIC | 964.8 | 673.0 |
| BIC | 1086.8 | 795.0 |

*Abbreviations:* β, unstandardized regression coefficient; AIC, Akaike information criterion; BIC, Bayesian information criterion; BMI, body mass index; CI, confidence interval; DDD, degenerative disc disease; RMSE, root mean square error. *Notes:* Full models adjusted for all variables shown. EQ-5D-5L ^cw^ index derived using crosswalk algorithm. EQ-5D-3L index calculated using Singapore value set. Presentation pathway at registry recruitment indicates the route by which patients entered the spine care pathway at recruitment: outpatient clinic vs non-outpatient presentation (emergency department or direct inpatient recruitment). Results are consistent across both indices, supporting robustness of findings. *p < 0.05; **p < 0.01; ***p < 0.001
