# Supplementary material for: Correction: Factors associated with preoperative health-related quality of life in patients undergoing lumbar spine surgery: a multi-ethnic Asian cohort
Source: Qual Life Res. 2026 Jul 13;35(8):230. doi: 10.1007/s11136-026-04304-x (PMC13364872; doi:10.1007/s11136-026-04304-x)
Supplement: Supplementary file 1 — Supplementary Material 1 [file 11136_2026_4304_MOESM1_ESM.docx]

**Replacement Supplementary Tables**

*Corrigendum to:* Li X, Chern CWJ, Teo AQA, Tan JHJ, Vasan Thakumar A, Luo N, Hey HWD, Cheng LJ. Factors associated with preoperative health-related quality of life in patients undergoing lumbar spine surgery: a multi-ethnic Asian cohort. *Quality of Life Research* 2026; 35:144. https://doi.org/10.1007/s11136-026-04257-1

**Contents**

Supplementary Table 2. Replacement: Sensitivity hierarchical linear regression on EQ-5D-3L index

Supplementary Table 3. Replacement: Comparison of full models, EQ-5D-5L crosswalk versus EQ-5D-3L

**Note**

All coefficients in this document were re-fitted from the original analytic dataset, spine_dataset.dta, using the same variable specifications, complete-case selection, and outcome variables: the EQ-5D-5L crosswalk index for the main analyses, the EQ-5D-3L index for Supplementary Table 2, and the EQ-5D-3L column of Supplementary Table 3. Model fit indices, including R², AIC, BIC, and AUC, match the published values exactly, confirming that the underlying numeric analyses are unchanged. Only the value labels for Sex, Race/ethnicity (Malay/Indian), and Education (cyclical rotation of Primary, Secondary, and Post-secondary/Diploma categories) have been corrected.

**Supplementary Table 2. Sensitivity hierarchical linear regression of predictors of EQ-5D-3L index**

| ***Characteristic*** | ***Model 1 β (95% CI)*** | ***Model 2 β (95% CI)*** | ***Model 3 β (95% CI)*** |
| --- | --- | --- | --- |
| **Socio-demographic** |  |  |  |
| Age category |  |  |  |
| Young adults (<45) | Ref | Ref | Ref |
| Middle-aged (45–64) | 0.03 (-0.02, 0.09) | 0.01 (-0.05, 0.07) | -0.01 (-0.07, 0.05) |
| Older adults (≥65) | 0.04 (-0.02, 0.09) | 0.00 (-0.07, 0.07) | -0.01 (-0.08, 0.05) |
| Sex |  |  |  |
| Male | Ref | Ref | Ref |
| Female | -0.04 (-0.08, -0.00) * | -0.04 (-0.08, -0.00) * | -0.05 (-0.09, -0.01) * |
| Race/ethnicity |  |  |  |
| Chinese | Ref | Ref | Ref |
| Malay | -0.14 (-0.20, -0.08) *** | -0.13 (-0.19, -0.06) *** | -0.09 (-0.15, -0.03) ** |
| Indian | -0.13 (-0.20, -0.06) *** | -0.13 (-0.20, -0.06) *** | -0.09 (-0.16, -0.03) ** |
| Others | -0.12 (-0.19, -0.05) *** | -0.12 (-0.18, -0.05) ** | -0.11 (-0.17, -0.04) ** |
| Education level |  |  |  |
| Primary or below | Ref | Ref | Ref |
| Secondary | 0.08 (0.03, 0.14) ** | 0.08 (0.03, 0.14) ** | 0.07 (0.02, 0.13) ** |
| Post-secondary/Diploma | 0.13 (0.07, 0.19) *** | 0.13 (0.07, 0.19) *** | 0.11 (0.06, 0.17) *** |
| University and above | 0.12 (0.06, 0.18) *** | 0.12 (0.06, 0.19) *** | 0.10 (0.04, 0.16) ** |
| **Clinical** |  |  |  |
| BMI category |  |  |  |
| Normal/Underweight (<23) | — | Ref | Ref |
| Overweight (23–27.4) | — | 0.03 (-0.02, 0.08) | 0.03 (-0.02, 0.07) |
| Obese (≥27.5) | — | -0.02 (-0.07, 0.03) | -0.02 (-0.07, 0.03) |
| Comorbidity status |  |  |  |
| No comorbidities | — | Ref | Ref |
| ≥1 comorbidity | — | -0.01 (-0.06, 0.04) | -0.02 (-0.06, 0.03) |
| Diagnosis |  |  |  |
| Spinal stenosis | — | Ref | Ref |
| Prolapsed intervertebral disc | — | -0.06 (-0.12, 0.00) | -0.04 (-0.10, 0.02) |
| Spondylolisthesis | — | -0.00 (-0.05, 0.05) | -0.01 (-0.06, 0.04) |
| DDD | — | -0.02 (-0.07, 0.04) | -0.02 (-0.07, 0.04) |
| Spine level involvement |  |  |  |
| L4/5 | — | Ref | Ref |
| L4/5 and L5/S1 | — | 0.00 (-0.06, 0.07) | 0.02 (-0.04, 0.09) |
| L5/S1 | — | -0.04 (-0.10, 0.02) | -0.03 (-0.09, 0.02) |
| Mixed level | — | -0.06 (-0.14, 0.02) | -0.04 (-0.12, 0.03) |
| Others | — | -0.02 (-0.07, 0.03) | -0.01 (-0.05, 0.04) |
| **Healthcare and lifestyle** |  |  |  |
| Presentation pathway |  |  |  |
| Outpatient clinic | — | — | Ref |
| Non-outpatient presentation | — | — | -0.33 (-0.41, -0.26) *** |
| History of accident/trauma |  |  |  |
| No | — | — | Ref |
| Yes | — | — | -0.09 (-0.17, -0.01) * |
| Smoking history |  |  |  |
| Never smoker | — | — | Ref |
| Former smoker | — | — | -0.04 (-0.11, 0.03) |
| Current smoker | — | — | -0.06 (-0.12, -0.00) * |
| **Model fit indices** |  |  |  |
| R² | 0.052 | 0.063 | 0.128 |
| Adjusted R² | 0.045 | 0.048 | 0.111 |
| RMSE | 0.328 | 0.327 | 0.316 |
| AIC | 732.2 | 737.9 | 660.5 |
| BIC | 783 | 839.6 | 782.5 |

*Abbreviations: β, unstandardised regression coefficient; AIC, Akaike information criterion; BIC, Bayesian information criterion; BMI, body mass index; CI, confidence interval; DDD, degenerative disc disease; RMSE, root mean square error. Notes: Outcome: EQ-5D-3L index. Significance levels: *p<0.05; **p<0.01; ***p<0.001.*

**Supplementary Table 3. Comparison of full-model results: EQ-5D-5L crosswalk index versus EQ-5D-3L index**

| ***Characteristic*** | ***EQ-5D-5L crosswalk β (95% CI)*** | ***EQ-5D-3L β (95% CI)*** |
| --- | --- | --- |
| **Socio-demographic** |  |  |
| Age category |  |  |
| Young adults (<45) | Ref | Ref |
| Middle-aged (45–64) | -0.00 (-0.07, 0.06) | -0.01 (-0.07, 0.05) |
| Older adults (≥65) | -0.02 (-0.09, 0.06) | -0.01 (-0.08, 0.05) |
| Sex |  |  |
| Male | Ref | Ref |
| Female | -0.06 (-0.10, -0.01) * | -0.05 (-0.09, -0.01) * |
| Race/ethnicity |  |  |
| Chinese | Ref | Ref |
| Malay | -0.08 (-0.15, -0.01) * | -0.09 (-0.15, -0.03) ** |
| Indian | -0.10 (-0.17, -0.02) * | -0.09 (-0.16, -0.03) ** |
| Others | -0.12 (-0.20, -0.05) ** | -0.11 (-0.17, -0.04) ** |
| Education level |  |  |
| Primary or below | Ref | Ref |
| Secondary | 0.10 (0.04, 0.16) ** | 0.07 (0.02, 0.13) ** |
| Post-secondary/Diploma | 0.15 (0.09, 0.21) *** | 0.11 (0.06, 0.17) *** |
| University and above | 0.16 (0.09, 0.23) *** | 0.10 (0.04, 0.16) ** |
| **Clinical** |  |  |
| BMI category |  |  |
| Normal/Underweight (<23) | Ref | Ref |
| Overweight (23–27.4) | 0.03 (-0.03, 0.08) | 0.03 (-0.02, 0.07) |
| Obese (≥27.5) | -0.03 (-0.09, 0.02) | -0.02 (-0.07, 0.03) |
| Comorbidity status |  |  |
| No comorbidities | Ref | Ref |
| ≥1 comorbidity | -0.03 (-0.08, 0.02) | -0.02 (-0.06, 0.03) |
| Diagnosis |  |  |
| Spinal stenosis | Ref | Ref |
| Prolapsed intervertebral disc | -0.03 (-0.10, 0.04) | -0.04 (-0.10, 0.02) |
| Spondylolisthesis | 0.00 (-0.06, 0.06) | -0.01 (-0.06, 0.04) |
| DDD | -0.03 (-0.09, 0.03) | -0.02 (-0.07, 0.04) |
| Spine level involvement |  |  |
| L4/5 | Ref | Ref |
| L4/5 and L5/S1 | 0.04 (-0.03, 0.11) | 0.02 (-0.04, 0.09) |
| L5/S1 | -0.04 (-0.10, 0.03) | -0.03 (-0.09, 0.02) |
| Mixed level | -0.05 (-0.13, 0.04) | -0.04 (-0.12, 0.03) |
| Others | 0.00 (-0.05, 0.06) | -0.01 (-0.05, 0.04) |
| **Healthcare and lifestyle** |  |  |
| Presentation pathway |  |  |
| Outpatient clinic | Ref | Ref |
| Non-outpatient presentation | -0.37 (-0.46, -0.28) *** | -0.33 (-0.41, -0.26) *** |
| History of accident/trauma |  |  |
| No | Ref | Ref |
| Yes | -0.11 (-0.20, -0.01) * | -0.09 (-0.17, -0.01) * |
| Smoking history |  |  |
| Never smoker | Ref | Ref |
| Former smoker | -0.05 (-0.13, 0.03) | -0.04 (-0.11, 0.03) |
| Current smoker | -0.07 (-0.13, -0.00) * | -0.06 (-0.12, -0.00) * |
| **Model fit indices** |  |  |
| R² | 0.132 | 0.128 |
| Adjusted R² | 0.115 | 0.111 |
| AIC | 964.8 | 660.5 |
| BIC | 1086.8 | 782.5 |

*Abbreviations: β, unstandardised regression coefficient; AIC, Akaike information criterion; BIC, Bayesian information criterion; BMI, body mass index; CI, confidence interval; DDD, degenerative disc disease. Notes: Full models adjusted for all variables shown. The EQ-5D-5L crosswalk index is derived using the van Hout 2021 crosswalk algorithm; the EQ-5D-3L index is calculated using the published value set. Results are consistent across both indices, supporting robustness of findings. Significance levels: *p<0.05; **p<0.01; ***p<0.001.*
